# Supplementary material for: Tricalbins Contribute to Cellular Lipid Flux and Form Curved ER-PM Contacts that Are Bridged by Rod-Shaped Structures
Source: Dev Cell. 2019 Nov 18;51(4):488–502.e8. doi: 10.1016/j.devcel.2019.09.019 (PMC6863393; doi:10.1016/j.devcel.2019.09.019)
Supplement: Document S1. Figures S1–S5 [file mmc1.pdf]

Developmental Cell, Volume *51*

Supplemental Information

Tricalbins Contribute to Cellular Lipid Flux and Form Curved ER-PM Contacts that Are Bridged by Rod-Shaped Structures

Patrick C. Hoffmann, Tanmay A.M. Bharat, Michael R. Wozny, Jerome Boulanger, Elizabeth A. Miller, and Wanda Kukulski

Supplemental Figure S1. Related to Figure 3.

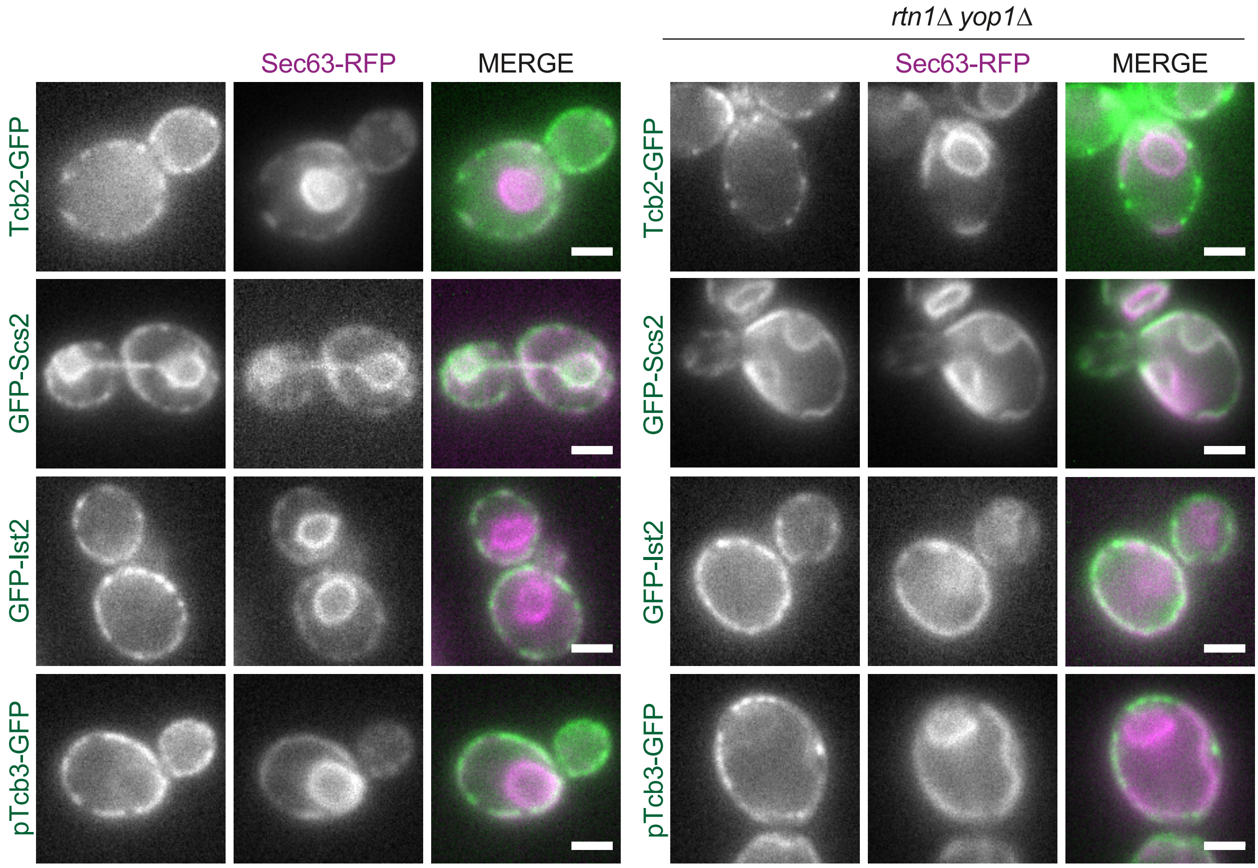

Supplemental Figure S1. Related to Figure 3. Reticulon-dependent localization of ER-PM bridging proteins

Live FM of wild type (left panel) and *rtn1*Δ *yop1*Δ (right panel) cells expressing either Tcb2-EFGP, GFP-Scs2, GFP-Ist2 or plasmid-encoded Tcb3-GFP, in combination with plasmid-encoded Sec63-RFP. Scale bars: 2 μm.

Supplemental Figure S2. Related to Figure 4.

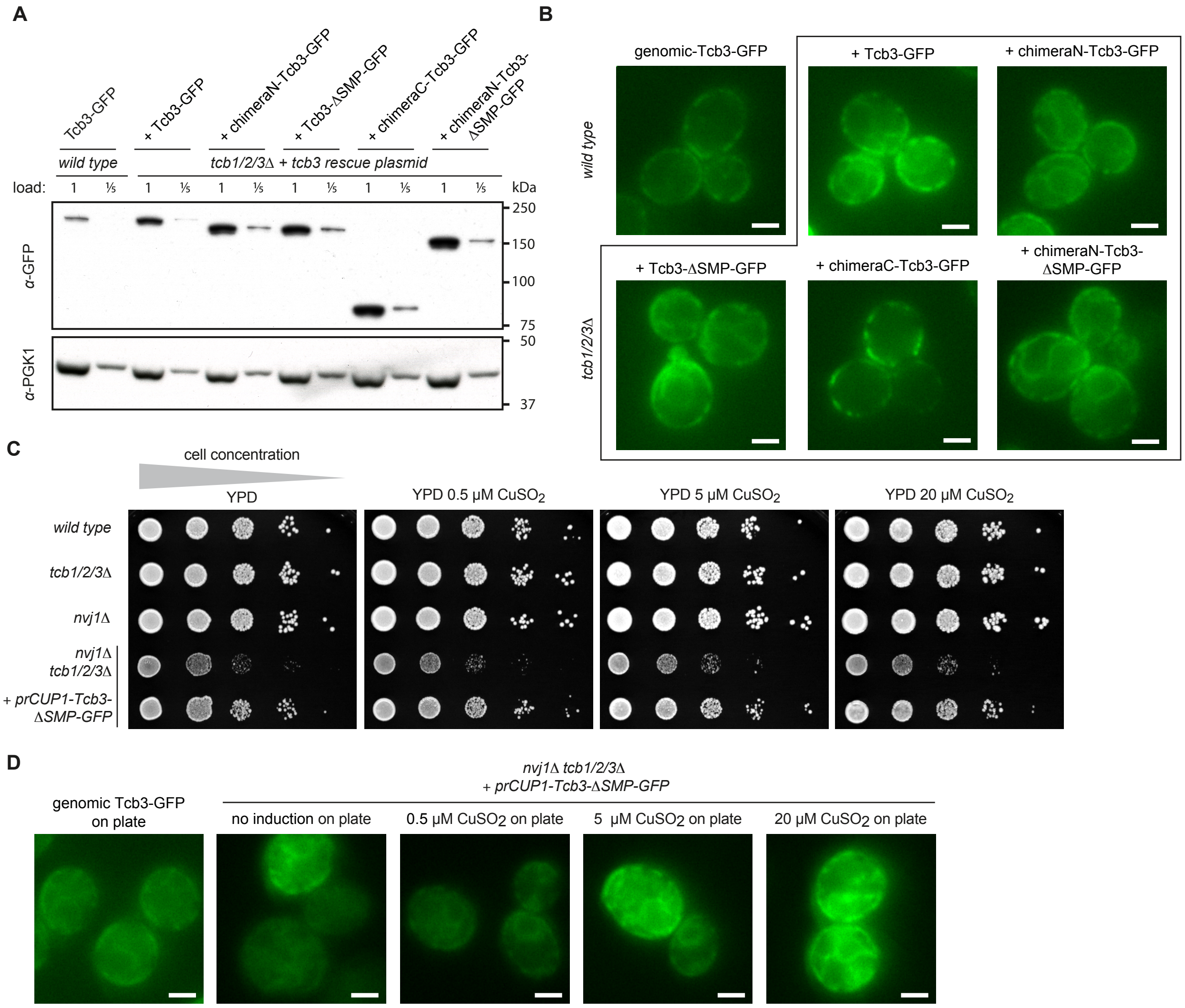

Supplemental Figure S2. Related to Figure 4. Protein expression levels of the rescue constructs used in the SGA

A: Western blot using an antibody against GFP, to compare levels of GFP-tagged Tcb3 at the genomic locus in wild type cells, with the different GFP-tagged Tcb3 rescue plasmids (see Figure 4) in the *tcb1/2/3*Δ query strain. For each sample 30 µg total protein (lanes labeled 1) and a five-fold dilution (lanes labeled 1/5) was loaded. Lower panel: Pgk1 was blotted for as loading control. **B:** Live FM of GFP-tagged Tcb3 at the genomic locus in wild type cells, and of the different GFP-tagged Tcb3 rescue plasmids in the *tcb1/2/3*Δ query strain, using the same imaging conditions. **C:** Serial dilution growth assays comparing growth of the *tcb1/2/3*Δ query strain to single *nvj1*Δ and quadruple *nvj1*Δ *tcb1/2/3*Δ deletions, without and with copper-titratable *prCUP1-Tcb3-*Δ*SMP-GFP* rescue plasmid on substrates with varying $CuSO_2$ concentrations. **D:** Live FM of cells expressing Tcb3-GFP from the endogenous locus and of *nvj1*Δ *tcb1/2/3*Δ cells with *prCUP1-Tcb3-*Δ*SMP-GFP* after 2 days of growth on plates with varying $CuSO_2$ concentrations, thus comparable to the serial dilution growth assays. Fluorescent images in B and D are adjusted to the same contrast and brightness levels. Scale bars: 2 *µ*m in B and D.

Supplemental Figure S3. Related to Figure 5.

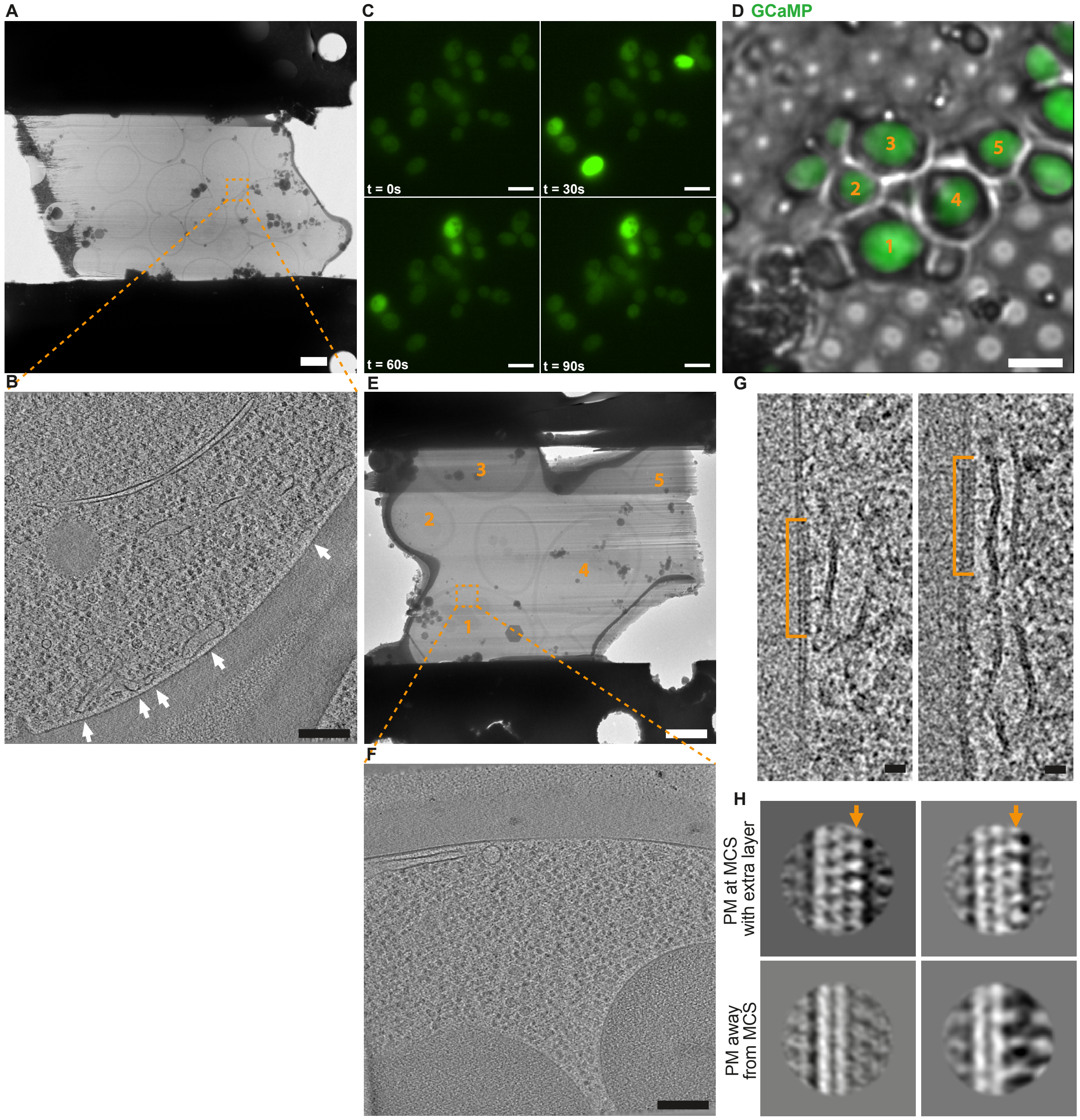

Supplemental Figure S3. Related to Figure 5. Cryo-CLEM for targeting cells with high cytosolic Ca^{2+} by cryo-FIB milling and cryo-ET

A: Overview cryo-EM image of a lamella prepared from untreated *scs2/22*Δ *ist2*Δ cells. Orange dashed square indicates the field of view of the electron cryo-tomogram shown in B. **B:** Virtual slice through electron cryo-tomogram acquired at the lamella position indicated in A. Left image in Figure 5A is from the same electron cryo-tomogram. White arrows indicate tubular cER cisternae. **C:** Live FM of a group of *scs2/22*Δ *ist2*Δ cells expressing GCaMP. GCaMP signal (green) imaged at different time points (0s, 30s, 60s and 90s) after exposure to 200 mM $CaCl_2$. **D:** Cryo-FM image of *scs2/22*Δ *ist2*Δ cells expressing GCaMP; overlay of green and bright field signals. Cells that display strong GCaMP signals, and that are visible in the cryo-FIB milled lamella (E) prepared from this group of cells, are labeled by matching numbers 1 – 5 in both D and E. **E:** Cryo-EM overview of FIB-milled lamella generated from the group of cells shown in D; labeled by matching numbers. Orange dashed square indicates the field of view of the electron cryo-tomogram shown in F. Note that this image corresponds to the image in Figure 5B, but is shown here without overlaid GCaMP signal. **F:** Virtual slice through electron cryo-tomogram acquired at the lamella position indicated above in cell number 1. Magnified views of virtual slices from this tomogram are shown in Figure 5C left and middle. **G:** Virtual slices of electron cryo-tomograms of cells displaying strong GCaMP signals, examples showing an extra density layer (orange brackets) in addition to those shown in Figure 5C. Left panel is a different slice from the same tomogram as shown in middle panel of Supplemental Figure S4A. **H:** 2D class averages from subtomogram averaging of PM within ER-PM contact with extra layer (top images), and PM outside of ER-PM contact (bottom). Class averages to the left and right correspond to tomogram shown in left and right image of G, respectively. In class average images, extracellular PM leaflets are facing left, cytosolic leaflets are facing right. Scale bars: 2 μm in A and E, 200 nm in B and F, 5 μm in C and D, and 20 nm in G.

Supplemental Figure S4. Related to Figures 5 and 6.

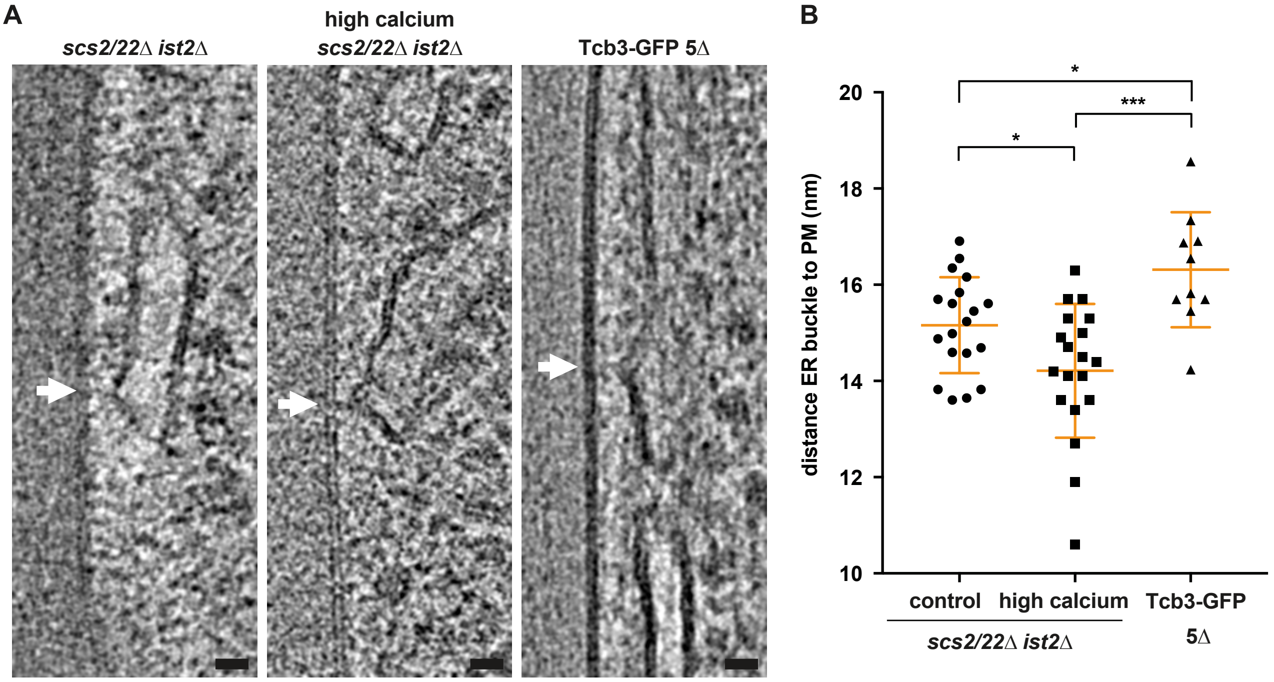

Supplemental Figure S4. Related to Figures 5 and 6. The cER locally buckles towards the plasma membrane

A: Virtual slices through electron cryo-tomograms of three different conditions; untreated *scs2/22*Δ *ist2*Δ cells, Ca^{2+}-treated *scs2/22*Δ *ist2*Δ cells displaying strong GCaMP signal, and 5Δ cells expressing galactose-induced Tcb3-GFP. Arrows indicate local buckles of the cER. Middle panel is a different slice through the same tomogram as shown in left panel of Supplemental Figure S3G. Scale bars: 20 nm. **B:** Distances between the buckling cER and the plasma membrane, measured locally at positions of buckling. Orange lines represent mean and SD (untreated vs. treated: P=0.0213, untreated vs. 5Δ cells: P=0.0192, treated vs. 5Δ cells: P=0.0004).

Supplemental Figure S5. Related to Figure 6.

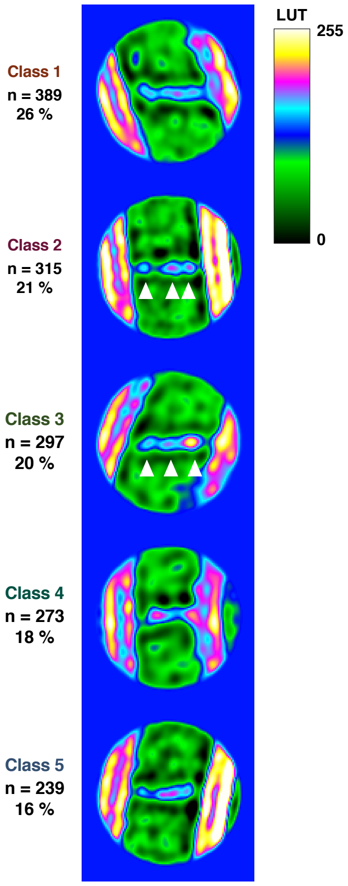

Supplemental Figure S5. Related to Figure 6. 2D class averages reveal linear density organization of rod-like particles

2D class averages of subvolumes containing bridging particles as shown in Figure 6F, but the grey scale was converted to heat map for better visibility of density variations, indicated by arrowheads in classes 2 and 3. LUT indicates color corresponding to gray scale values. Number of particles per class (n), as well as percentage of total particle set, are indicated as in Figure 6F.
