# Supplementary material for: Tricalbins Contribute to Cellular Lipid Flux and Form Curved ER-PM Contacts that Are Bridged by Rod-Shaped Structures
Source: Dev Cell. 2019 Nov 18;51(4):488–502.e8. doi: 10.1016/j.devcel.2019.09.019 (PMC6863393; doi:10.1016/j.devcel.2019.09.019)
Supplement: Document S2. Article plus Supplemental Information [file mmc4.pdf]

Article

Developmental Cell

Tricalbins Contribute to Cellular Lipid Flux and Form Curved ER-PM Contacts that Are Bridged by Rod-Shaped Structures

Graphical Abstract

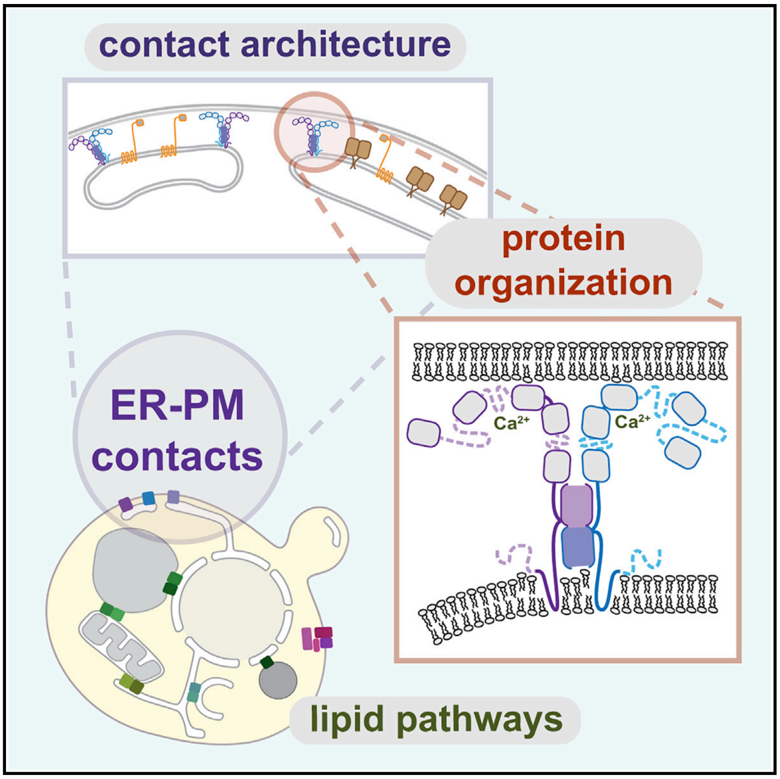

Authors

Patrick C. Hoffmann, Tanmay A.M. Bharat, Michael R. Wozny, Jerome Boulanger, Elizabeth A. Miller, Wanda Kukulski

Correspondence

kukulski@mrc-lmb.cam.ac.uk

In Brief

Hoffmann et al. investigate the architecture and function of yeast ER-PM contact sites. They show how bridging proteins, particularly tricalbins, relate to membrane curvature and inter-organelle distance. Genetic screening points to functional overlap between tricalbins and multiple lipid distribution pathways. Cryo-ET visualizes rod-shaped linkers at tricalbin-mediated ER-PM contacts.

Highlights

- ER-PM proteins form contact sites with distinct ER shape but similar distance
- Tricalbins localize to high ER curvature via their membrane domain
- Genetic screens link tricalbin function to pathways for cellular lipid distribution
- Cryo-ET reveals rod-shaped densities at tricalbin-mediated ER-PM contacts

Hoffmann et al., 2019, Developmental Cell *51*, 488–502
November 18, 2019 © 2019 MRC Laboratory of Molecular Biology.
Published by Elsevier Inc.
https://doi.org/10.1016/j.devcel.2019.09.019

CellPress

CellPress

Developmental Cell
Article

Tricalbins Contribute to Cellular Lipid Flux and Form Curved ER-PM Contacts that Are Bridged by Rod-Shaped Structures

Patrick C. Hoffmann,[1] Tanmay A.M. Bharat,[2,3] Michael R. Wozny,[1] Jerome Boulanger,[1] Elizabeth A. Miller,[1] and Wanda Kukulski[1,4,*]
[1]Cell Biology Division, MRC Laboratory of Molecular Biology, Francis Crick Avenue, Cambridge CB2 0QH, UK
[2]Sir William Dunn School of Pathology, University of Oxford, South Parks Road, Oxford OX1 3RE, UK
[3]Central Oxford Structural Microscopy and Imaging Centre, South Parks Road, Oxford OX1 3RE, UK
[4]Lead Contact
*Correspondence: kukulski@mrc-lmb.cam.ac.uk
https://doi.org/10.1016/j.devcel.2019.09.019

SUMMARY

Lipid flow between cellular organelles occurs via membrane contact sites. Extended-synaptotagmins, known as tricalbins in yeast, mediate lipid transfer between the endoplasmic reticulum (ER) and plasma membrane (PM). How these proteins regulate membrane architecture to transport lipids across the aqueous space between bilayers remains unknown. Using correlative microscopy, electron cryo-tomography, and high-throughput genetics, we address the interplay of architecture and function in budding yeast. We find that ER-PM contacts differ in protein composition and membrane morphology, not in intermembrane distance. *In situ* electron cryo-tomography reveals the molecular organization of tricalbin-mediated contacts, suggesting a structural framework for putative lipid transfer. Genetic analysis uncovers functional overlap with cellular lipid routes, such as maintenance of PM asymmetry. Further redundancies are suggested for individual tricalbin protein domains. We propose a modularity of molecular and structural functions of tricalbins and of their roles within the cellular network of lipid distribution pathways.

INTRODUCTION

The endoplasmic reticulum (ER) forms a membrane network of thin tubules, densely reticulated regions, and extended sheet-like cisternae (Nixon-Abell et al., 2016; West et al., 2011). This network interacts with virtually all other organelles through membrane contact sites (MCSs) that mediate metabolite exchange and signaling (Valm et al., 2017). At the cell periphery, the ER is in contact with the plasma membrane (PM) by close apposition of 10–30 nm (Fernández-Busnadiego et al., 2015; West et al., 2011). Such ER-PM contacts provide spatial control over Ca^{2+} regulation and signaling and mediate key transfer steps in lipid metabolism (Saheki and De Camilli, 2017). In budding yeast, about one-third of the PM is covered by ER (Loewen et al., 2007), which is therefore often referred to as cortical ER (cER). The vast majority of yeast ER-PM contacts are mediated by a set of six proteins from three families, which are conserved between yeast and humans: two VAMP-associated proteins (VAPs), Scs2 and Scs22; TMEM16-like Ist2; and three Extended-Synaptotagmin (E-Syt) orthologs, called tricalbins Tcb1, Tcb2, and Tcb3 (Manford et al., 2012). All six proteins are integral to the ER membrane and bind to the PM by interacting with lipid head groups or other proteins. The proteins of the three families differ in domain organization as well in the length of the putative linkers bridging the contact site (Gatta and Levine, 2016).

Besides their structural role in membrane bridging, these proteins contribute functionality to the ER-PM interface. The VAPs are interaction hubs for more than 100 proteins, many of which are lipid transfer proteins (LTPs) specific for MCSs (Loewen et al., 2003; Murphy and Levine, 2016). The TMEM16 family consists of Cl^- channels and lipid scramblases (Brunner et al., 2014; Suzuki et al., 2010). E-Syts are directly capable of transferring lipids between membranes (Saheki et al., 2016; Yu et al., 2016), and absence of E-Syts or yeast tricalbins leads to impaired control of PM homeostasis (Bian et al., 2018; Omnus et al., 2016). Similar to E-Syts, tricalbins contain a synaptotagmin-like mitochondrial-lipid-binding protein (SMP) domain as well as four or five C2 domains (Giordano et al., 2013; Kopec et al., 2010; Schulz and Creutz, 2004; Toulmay and Prinz, 2012). The SMP domain harbors lipids and is required for *in vitro* lipid transfer by E-Syts, while at least some of the C2 domains bind to the phosphoinositide $PI(4,5)P_2$ in the PM in a Ca^{2+}-dependent manner (Bian et al., 2018; Giordano et al., 2013; Saheki et al., 2016; Schauder et al., 2014; Schulz and Creutz, 2004).

ER-PM contact sites thus have complex macromolecular architectures with diverse components that contribute to multiple cellular processes. Protein organization and function are profoundly coupled at these sites, yet detailed understanding on the interplay between protein structure, membrane architecture, and contact site function is lacking. Furthermore, the contributions of individual contact site proteins to cell physiology remain difficult to assess, likely due to redundancies (Saheki and De Camilli, 2017; Wong et al., 2017). We have combined correlative light

 © 2019 MRC Laboratory of Molecular Biology. Published by Elsevier Inc.
This is an open access article under the CC BY license (http://creativecommons.org/licenses/by/4.0/).

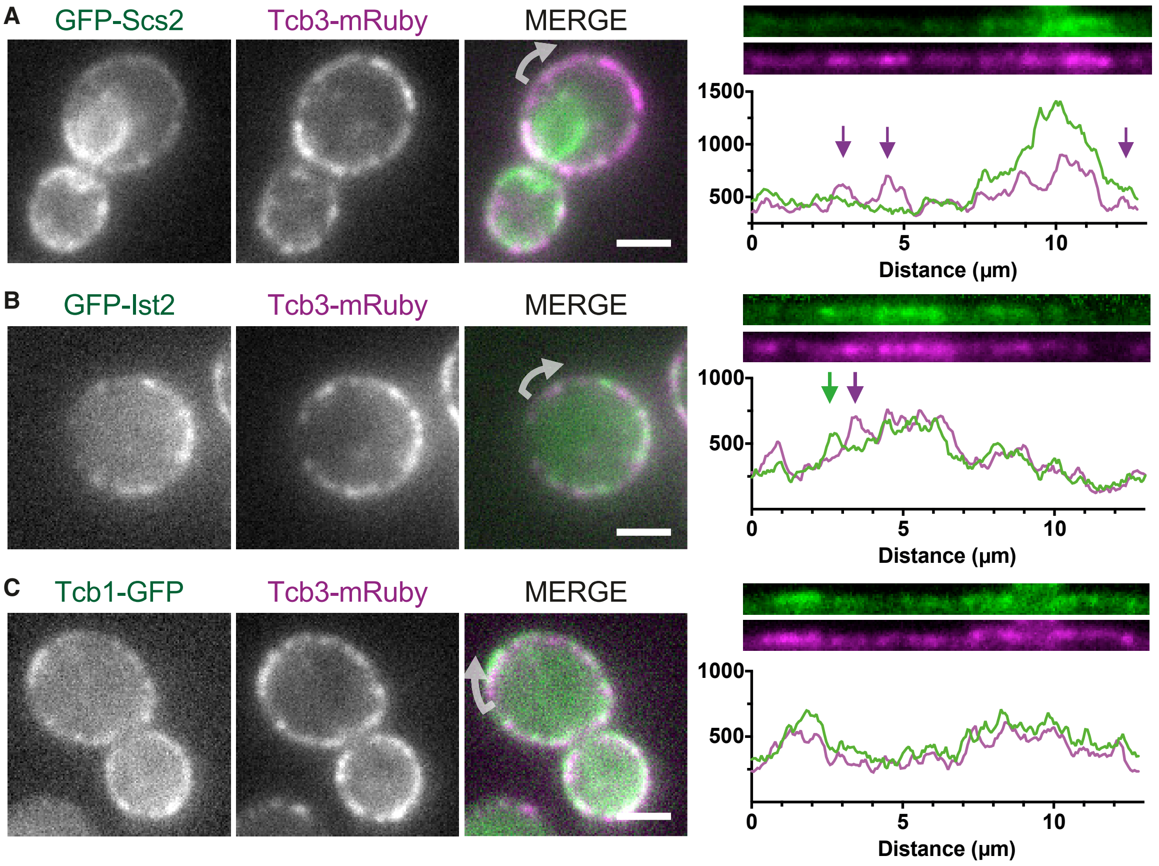

Figure 1. Proteins Mediating ER-PM Contacts Are Not Distributed Homogenously across the cER

Live FM of yeast cells expressing Tcb3-mRuby in combination with either (A) GFP-Scs2, (B) GFP-Ist2, or (C) Tcb1-GFP. All proteins are expressed from their endogenous genomic loci. In the merge of the two channels, arrows indicate the starting point of the linearized signals along the mother cell cortex, shown in the right panels, which also show the line profiles along linearized signals (pixel intensity in arbitrary units). Arrows indicate regions in which the signals differ. Scale bars, 2 µm.

and electron microscopy (CLEM), electron cryo-tomography (cryo-ET) of cryo-focused ion beam (cryo-FIB)-milled cells, and live-cell imaging with high content yeast genetics to unravel the intricate relationship between structure and function of ER-PM contact sites in budding yeast.

RESULTS

ER-PM Proteins Are Distributed Non-homogenously within the cER

We first investigated whether the protein families mediating ER-PM contacts are distributed equally throughout the cER. We imaged by live fluorescence microscopy (FM) yeast cells in which we chromosomally tagged pairs of bridging proteins with fluorescent proteins (Figure 1). By pairing one protein from each family with the most abundant tricalbin Tcb3, we aimed to compare the distributions of different families as well as among tricalbins. All tagged proteins localized to cER as described (Loewen and Levine, 2005; Manford et al., 2012; Toulmay and Prinz, 2012; Wolf et al., 2012). We analyzed the degree of colocalization among the different pairs by plotting fluorescence intensity profiles along the cell cortex. The paired profiles of Tcb3-mRuby and GFP-Scs2, as well as of Tcb3-mRuby and GFP-Ist2, overlapped extensively, indicating colocalization within most of the cER (Figures 1A and 1B). Remarkably, in both cases, the paired profiles did not completely overlap. Individual peaks of intensity indicated regions at which either of the proteins was enriched relative to the other. In contrast, the intensity profile of Tcb1-GFP overlapped completely with Tcb3-mRuby (Figure 1C). These data indicate that the distribution of different protein families within the cER is not homogeneous.

ER-PM Proteins Associate with Distinct ER Shapes but Similar Intermembrane Distances

We next asked whether the differential distribution could be linked to differences in ER-PM ultrastructure. We reasoned that local accumulation of individual proteins within the cER might generate functionally specific environments by modulating the ultrastructure. We applied CLEM on resin-embedded yeast cells (Kukulski et al., 2011). In electron tomograms of the locations of GFP signals, we visualized the 3D membrane architecture of the ER and the PM correlated with the presence of GFP-Scs2, GFP-Ist2, or Tcb3-GFP (Figures 2A–2C). In these tomograms, we also found regions of cER that were not

CellPress

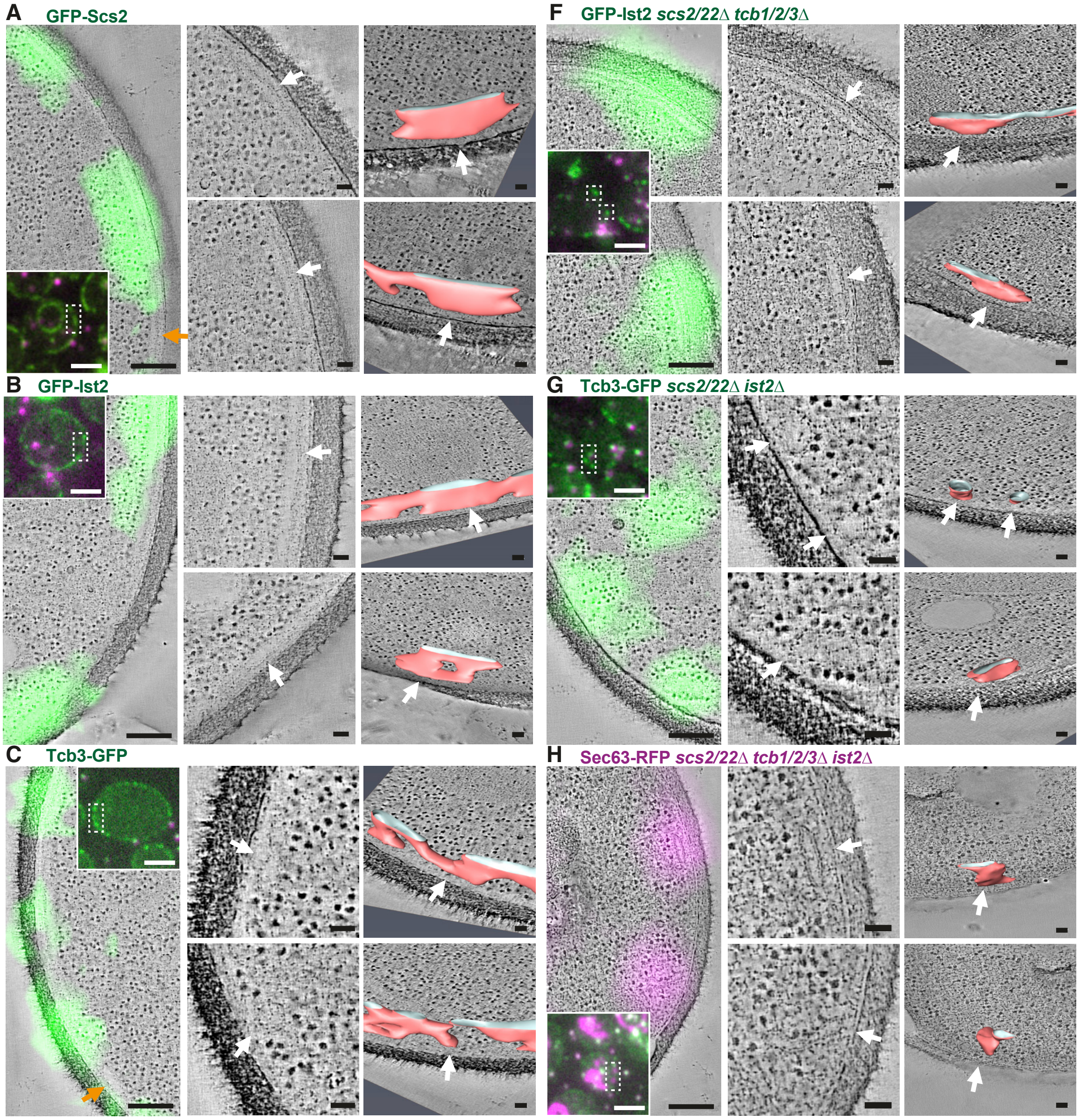

D

	# of observations	flat ER sheet	tubular ER	mixed flat & tubular	not assigned
GFP-Scs2	35	51 %	31 %	6 %	11 %
GFP-Ist2	42	36 %	29 %	24 %	12 %
Tcb3-GFP	46	20 %	50 %	26 %	4 %
GFP-Ist2 *scs2/22Δ tcb1/2/3Δ*	63	24 %	52 %	22 %	2 %
Tcb3-GFP *scs2/22Δ ist2Δ*	46	2 %	85 %	2 %	11 %
Sec63-RFP *scs2/22Δ tcb1/2/3Δ ist2Δ*	17	29 %	35 %	35 %	0 %

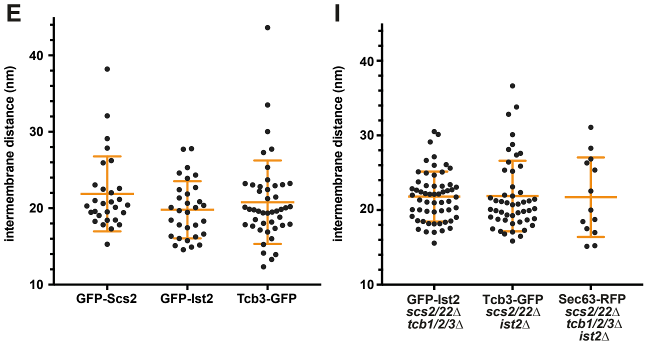

Figure 2. ER-PM Proteins Associate with Distinct ER Shapes but Mediate Similar Intermembrane Distances

(A–C and F–H) CLEM of resin-embedded yeast cells, genotype indicated above panels. Insets: FM of resin sections of wild-type (A)–(C) and mutant (F)–(H) cells expressing GFP-tagged bridging proteins (green) or Sec63-RFP (magenta). Fiducial markers are visible in magenta and green. Left images in (A)–(C) and (F)–(H) are virtual slices through electron tomograms, overlaid with GFP or RFP signals transformed according to fiducial-based correlation. Middle panels are magnified views of the same virtual slices, depicting the ER associated with GFP or RFP signals. Arrows indicate matching positions with images in the right panels, which

(legend continued on next page)

correlated with GFP signals, indicating absence or very low levels of the GFP-tagged protein (Figures 2A and 2C, orange arrows). These results corroborate that bridging proteins of different families are distributed non-homogenously within the cER. Moreover, some of the cER regions devoid of the protein of interest were immediately continuous with cER regions containing the protein of interest, indicating that bridging proteins may also segregate within individual cER subcompartments.

We classified the observed cER shapes into sheet-like flat ER cisternae, tubular ER of high membrane curvature, and ER consisting of both flat and tubular segments (mixed curvature) (Figure 2D). GFP-Scs2 and GFP-Ist2 predominantely associated with flat cER sheets (51% and 36%, respectively, of individual fluorescent signals, referred to as observations). Tcb3-GFP localized predominantly to tubular cER (50% of observations). At sites associated with the presence of the GFP-tagged proteins, we measured the distances between the cytosolic leaflets of the PM-facing ER membrane and the PM (see STAR Methods) and found that they were similar (Figure 2E). The average distance for GFP-Scs2 containing contact sites was 21.9 nm (SD 4.9 nm, N = 29 sites in 14 cells), for GFP-Ist2 19.8 nm (SD 3.7 nm, N = 31 sites in 12 cells), and for Tcb3-GFP 20.8 nm (SD 5.5 nm, N = 45 sites in 17 cells).

Given the high degree of colocalization between the different bridging proteins (Figure 1), the observed cER architectures likely contained not only the GFP-tagged protein but also some or all of the other bridging proteins. To assess the impact of the different proteins separately, we applied CLEM to cells expressing only one type of bridging protein. In the absence of VAPs and tricalbins (*scs2/22Δ tcb1/2/3Δ* cells), GFP-Ist2 was associated more often with tubular cER (52% of observations) than with sheets (24% of observations) (Figures 2D and 2F). Tcb3-GFP-containing cER in the absence of VAPs and Ist2 (*scs2/22Δ ist2Δ* cells) was predominantly tubular (85% of observations) (Figures 2D and 2G). The intermembrane distances were similar for Tcb3-GFP in *scs2/22Δ ist2Δ* cells (average 21.9 nm, SD 4.7 nm, N = 49 sites in 19 cells) and for GFP-Ist2 in *scs2/22Δ tcb1/2/3Δ* cells (average 21.8 nm, SD 3.3 nm, N = 57 sites in 24 cells) (Figure 2I).

Deletion of the six major ER-PM proteins does not completely abolish cER, and sterol transporters acting at ER-PM contacts may also have a bridging function (Gatta et al., 2015; Manford et al., 2012; Quon et al., 2018) We located the sparse residual cER in *scs2/22Δ ist2Δ tcb1/2/3Δ* cells by CLEM, using a plasmid-encoded broad ER marker, Sec63-RFP (Figure 2H) (Metzger et al., 2008). The cER in *scs2/22Δ ist2Δ tcb1/2/3Δ* cells had no preferred morphology (Figure 2D), and the intermembrane distances were similar to those in other mutant cells (average 21.7 nm, SD 5.3 nm, N = 13 sites in 10 cells) (Figure 2I).

These results show that the distribution of different bridging proteins correlates with differences in cER shape. In particular, Tcb3 displays a distinct preference for tubular cER of high membrane curvature. In contrast, the average intermembrane distances mediated by different bridging proteins are very similar. Taken together, these data indicate that differences in bridging protein composition of ER-PM contacts translate into differences in cER membrane curvature rather than differences in inter-organelle distance.

The Curvature Preference of Tricalbins Is Dictated by Their Membrane Domain

We next investigated the preference of tricalbins for high membrane curvature. ER tubulation is facilitated by reticulons, and absence of these proteins results in almost complete lack of tubular ER (Voeltz et al., 2006; West et al., 2011). Reticulons mediate membrane curvature through a hairpin structure inserted into the ER membrane (Hu et al., 2008; Voeltz et al., 2006). We asked how the distribution of Tcb3 depends on reticulons. In live cells, we imaged Tcb3-GFP in relation to plasmid-encoded Sec63-RFP. In wild-type cells, Tcb3-GFP and Sec63-RFP colocalized at the cER, albeit with some distinct areas of reduced correspondence (Figure 3A). In the absence of the two major reticulon-like proteins Rtn1 and Yop1 (Voeltz et al., 2006) (*rtn1Δ yop1Δ* cells), Tcb3-GFP and Sec63-RFP showed almost no colocalization. Although both proteins still localized to the cell cortex, Sec63-RFP formed extended sheet-like signals, as described (Voeltz et al., 2006), whereas Tcb3-GFP was exclusively punctate (Figure 3B). We observed the same localization pattern for Tcb2-GFP and plasmid-encoded Tcb3-GFP in combination with Sec63-RFP (Figure S1). In contrast, GFP-Ist2 and GFP-Scs2 entirely overlapped with Sec63-RFP in *rtn1Δ yop1Δ* cells (Figure S1). When we performed CLEM on Tcb3-GFP *rtn1Δ yop1Δ* cells, we found that Tcb3-GFP correlated almost exclusively to highly curved tubules at the cell cortex (Figures 3C and 3D). The same cells also contained extended cER sheets that were not associated with Tcb3-GFP (Figure 3E). These data indicate that the existence of highly curved cER tubules, which accommodate Tcb3-GFP, does not require the major reticulon-like proteins Rtn1 and Yop1.

To test the role of the Tcb3 membrane domain in its localization to curved cER, we generated a plasmid-encoded Tcb3-GFP construct in which we replaced the N-terminus including the predicted hairpin with the N-terminus of the ER-resident protein Erg11 including its single transmembrane alpha helix (Monk et al., 2014). We refer to this construct as chimeraN-Tcb3-GFP (Figure 3F). In *rtn1Δ yop1Δ* cells expressing plasmid-encoded Sec63-RFP, chimeraN-Tcb3-GFP colocalized extensively with Sec63-RFP in a sheet-like pattern, distinctive from the punctae formed by wild-type Tcb3-GFP (Figure 3G). These data indicate that the membrane domain of Tcb3 is responsible for localization of Tcb3-GFP to highly curved cER membranes.

These results provide further insight into the tricalbins' preference for curved membranes. In wild-type cells, tricalbins and the translocon component, Sec63, distribute within the cER shaped by reticulons. In the absence of the major reticulons, tricalbins

show virtual slices and segmentation models (rotated for better visibility; red, membrane and white, intersection at boundary of resin section) of the cER associated with GFP or RFP signals.

(D) Classification of cER morphologies associated with GFP or RFP signals in cells with different genotypes.

(E and I) Distances between cytosolic leaflets of the ER and PM, measured at contact sites associated with GFP or RFP signals. Orange lines indicate mean and SD.

Scale bars: 2 μm in FM images, 200 nm in FM-ET overlays, and 50 nm in ET and segmentation panels.

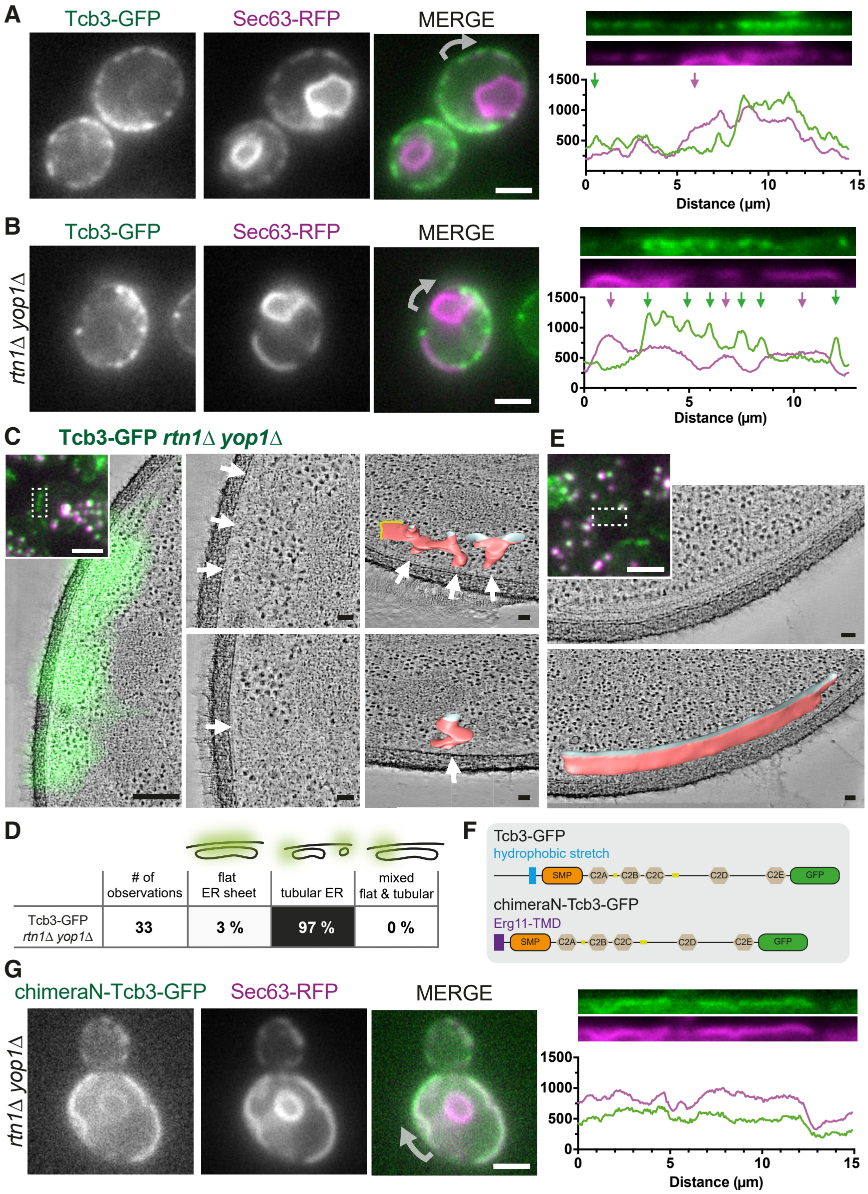

Figure 3. The ER Membrane Curvature Preference of Tcb3 Is Dictated by the Tcb3 Membrane Domain

(A and B) Live FM of wild-type (A) and *rtn1Δ yop1Δ* (B) yeast cells expressing Tcb3-GFP and Sec63-RFP. In the merge of the two channels, arrows indicate the starting point of the linearized signals along the mother cell cortex, shown in the right panels, which also show the line profiles along linearized signals (pixel intensity in arbitrary units). Arrows indicate regions in which the two signals differ.

(C and E) CLEM of resin-embedded *rtn1Δ yop1Δ* cells expressing Tcb3-GFP. Insets: FM of resin sections (GFP in green and fiducial markers in green and magenta). White dashed box corresponds to the area shown in the underlying virtual slice through electron tomogram in (C) overlaid with the Tcb3-GFP signal transformed according to fiducial-based correlation.

(C) Middle images are magnified views of the virtual slice shown in the left panel, depicting the cER associated with Tcb3-GFP. Arrows indicate matching positions with images in the right panel, which show virtual slices and segmentation models (rotated for better visibility; red, membrane; white, intersection at boundary of resin section; and yellow, segmentation not continued) of the cER associated with Tcb3-GFP.

(D) Classification of cER membrane morphology associated with Tcb3-GFP in *rtn1Δ yop1Δ* cells.

(E) Extended cER sheets devoid of Tcb3-GFP signals, lower panel shows segmentation model (rotated for better visibility; red: membrane, white: intersection at boundary of resin section).

(F) Tcb3-GFP domain structure. In chimeraN-Tcb3-GFP, the N-terminus (1–247 amino acids (aas)) including the membrane domain is replaced with the ER signal sequence and transmembrane domain (1–55 aas) of Erg11.

(G) Live FM of *rtn1Δ yop1Δ* cells expressing Sec63-RFP and chimeraN-Tcb3-GFP, both plasmid encoded. In the merge of the two channels, the arrow indicates the starting point of the linearized signals along the mother cell cortex, shown in the right panel, which also shows the line profile along linearized signals (pixel intensity in arbitrary units).

Scale bars: 2 μm in FM, 200 nm in FM-ET overlay, and 50 nm in ET and segmentations.

become restricted to subdomains of residual curvature, devoid of Sec63. The N-terminal membrane domain of Tcb3 is responsible for this segregation.

Tricalbins Functionally Interact with Pathways Involved in Lipid Sensing, Transfer, and Biosynthesis

Having defined a curvature localization domain of Tcb3, we next wanted to test the functional importance of this domain. Deletion of all three tricalbins causes no significant phenotype (Creutz et al., 2004; Manford et al., 2012; Toulmay and Prinz, 2012), possibly due to redundancies. Therefore, to reveal useful phenotypes that might be complemented by the curvature-insensitive form of Tcb3 (Figure 4A), we performed a synthetic genetic array (SGA) using a *tcb1/2/3Δ* query strain crossed to the haploid gene deletion and decreased abundance by mRNA perturbation (DAmP) collections (*genexΔ*) (Schuldiner et al., 2005; Tong et al., 2001) (Figure 4B). We scored growth phenotypes by measuring colony size of quadruple (*tcb1Δ tcb2Δ tcb3Δ genexΔ*) deletion mutants and calculating the $\log_2$ ratio relative to control strains (see STAR Methods). We considered a $\log_2$ ratio below -1, corresponding to a 50% reduction in colony size, to be a hit. About 700 genes showed negative synthetic interactions with *tcb1/2/3Δ* (Figure 4C; Table S1). Of those hits, about two-thirds displayed normal growth in *tcb1Δ tcb2Δ genexΔ* mutants (Table S1). We further considered those hits that were defective only in the absence of all three tricalbins and validated several of these hits using serial dilution growth assays (Figure 4D).

Among the hits, we identified many components involved in cellular lipid metabolism and membrane biology. We found genes encoding proteins previously characterized to function

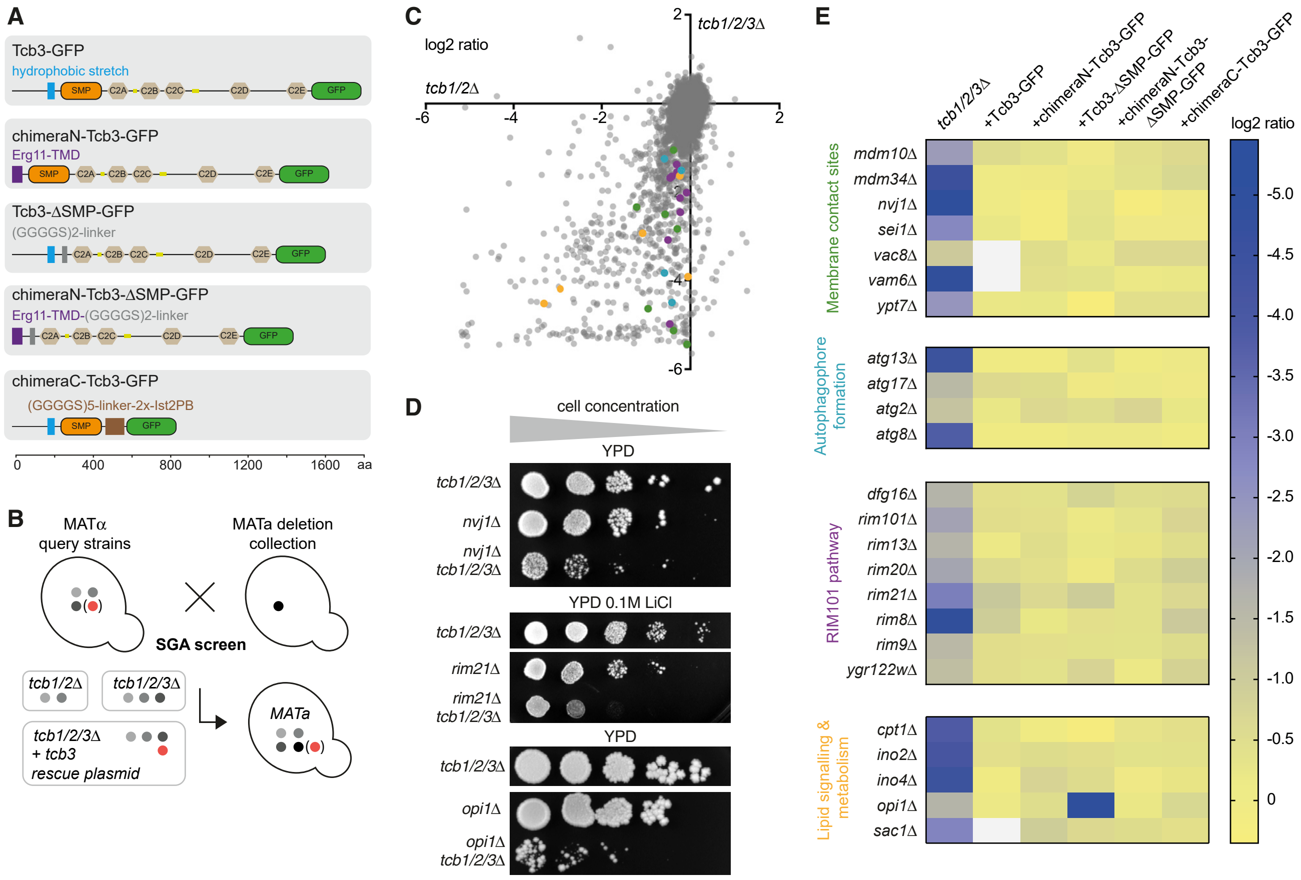

Figure 4. Synthetic Genetic Interactions of Tricalbins

(A) Sequence and domain structure of tricalbins and rescue constructs, which are plasmid-encoded wild-type Tcb3-GFP and mutants with N terminus including membrane domain (blue) replaced (purple) (chimeraN-Tcb3-GFP), SMP domain (orange) replaced (gray) (Tcb3-ΔSMP-GFP), both N terminus and SMP domain replaced (chimeraN-Tcb3-ΔSMP-GFP), or C2 domains (beige) replaced (brown). Yellow, putative coiled-coil regions.

(B) Schematic overview of SGA screen: the query strains, *tcb1/2Δ*, *tcb1/2/3Δ* or *tcb1/2/3Δ* with rescue plasmid were crossed to the yeast gene deletion and DAmP collections. Haploid mutants containing all deletion markers were selected on appropriate selection media. Colony size was scored and compared to mutants with random distribution of gene deletions.

(C) Plot of $\log_2$ ratios for negative genetic interactions of the *tcb1/2Δ* query strain (x axis) against the *tcb1/2/3Δ* query strain (y axis). Selected genes shown in (E) are highlighted in colors: membrane contact sites, green; autophagophore formation, blue; Rim101 pathway, purple; and lipid signaling and metabolism, orange.

(D) Validation of selected synthetic interactions by serial dilution growth assays on YPD or YPD with 0.1 M LiCl, comparing growth of the *tcb1/2/3Δ* query strain to single and quadruple deletions. Redundant lanes in the middle and the lower panels have been removed from original images, indicated by gaps.

(E) Heatmap of $\log_2$ ratios for negative genetic interactions of *tcb1/2/3Δ* (first column), grouped according to function. Second to sixth column are the rescues with the Tcb3 constructs depicted in (A).

at MCSs, including the nucleus-vacuole junction proteins Nvj1 and Vac8, and components of the ER-mitochondria encounter structure (ERMES) Mdm34 and Mdm10 (Figure 4E, top panel) (Kornmann et al., 2009; Pan et al., 2000). These hits suggest that tricalbin function is partially redundant with functions occurring at other MCSs, consistent with emerging models of lipid flow by parallel routes. We also identified genes involved in autophagy, such as *atg2*, which encodes an LTP suggested to be involved in autophagophore formation (Figure 4E, second panel) (Maeda et al., 2019; Osawa et al., 2019; Valverde et al., 2019). These hits suggest that tricalbin function can compensate for loss of lipid transfer to autophagosomal membranes. Finally, we found all core components of the RIM101 pathway (Figure 4E, third panel), which is involved in sensing alkaline pH and disturbance of PM lipid asymmetry (Ikeda et al., 2008; Lamb et al., 2001). These hits suggest a functional link between tricalbins and lipid distribution across the PM.

The Functional Interactions of Tricalbins Are Not Dependent on Their Major Protein Domains

Having identified phenotypic effects associated with tricalbin deletion, we next asked which genetic interactions could be rescued by expression of the plasmid-encoded chimeraN-Tcb3-GFP construct, the curvature-insensitive form of Tcb3, as compared to rescue by wild-type Tcb3-GFP, expressed from the same plasmid (Figures 3F and 4A). We found that chimeraN-Tcb3-GFP rescued most of the interactions that were rescued by wild-type Tcb3-GFP. In particular, all interactions with genes of the RIM101 pathway were rescued by chimeraN-Tcb3-GFP to a similar extent as by wild-type Tcb3-GFP.

Moreover, most interactions from the cohorts of MCS and autophagy genes were also rescued by chimeraN-Tcb3-GFP (Figure 4E).

The SMP domain is necessary for lipid transfer by E-Syts (Saheki et al., 2016; Yu et al., 2016) and is thus potentially also a major functional domain of tricalbins. To address its importance, we tested rescue of synthetic interactions in *tcb1/2/3*Δ cells by a construct in which we replaced the SMP domain in Tcb3-GFP by a linker sequence (Figure 4A). Similar to chimeraN-Tcb3-GFP, the plasmid-encoded ΔSMP construct rescued the majority of hits (Figure 4E).

As neither the SMP nor the membrane domain was required for rescue, we next tested if the two domains were redundant with each other. We generated a construct in which both were replaced (chimeraN-Tcb3-ΔSMP-GFP; Figure 4A). Similar to the single domain replacements, this construct also rescued the majority of hits (Figure 4E). This result indicates that both individually and in combination the SMP and membrane domains are functionally dispensable, suggesting additional roles for the remaining domain elements. We therefore tested whether the C-terminal C2 domains could provide functionality to Tcb3. We tested rescue with a construct in which we replaced all five C2 domains by a tandem repeat of the polybasic patch that recruits Ist2 to the PM (Manford et al., 2012) (chimeraC-Tcb3-GFP, Figure 4A). We found that this construct also rescued all hits, similar to the other constructs and to Tcb3-GFP (Figure 4E).

The plasmid-encoded constructs described above resulted in higher protein levels than when Tcb3-GFP was expressed from its endogenous locus (Figures S2A and S2B). The elevated protein levels might enhance rescue through compensatory effects. We therefore tested rescue of one of the hits, *nvj1*Δ, by the Tcb3-ΔSMP-GFP construct under the control of a titratable promoter. Growth of *nvj1*Δ *tcb1/2/3*Δ was rescued with levels of Tcb3-ΔSMP-GFP comparable to endogenously expressed Tcb3-GFP (Figures S2C and S2D), indicating that the function of the SMP domain is not compensated by excessive amounts of Tcb3-ΔSMP-GFP.

In summary, we have uncovered genetic interactions that indicate overlap of tricalbin function with specific cellular lipid distribution routes. Although these functions depend on the presence of at least one tricalbin in addition to VAPs and Ist2, they neither require this tricalbin to have its curvature sensitivity nor its SMP domain, which is presumed to mediate lipid transfer. Moreover, binding to the PM does not need to occur through the tricalbin C2 domains.

Cryo-ET Reveals Protein Densities at Tricalbin-Mediated Contact Sites

The structural organization of LTPs between two membranes is key to their function (Reinisch and De Camilli, 2016). We therefore visualized protein structures at tricalbin-mediated contacts using recent advances in cellular cryo-ET that allow imaging of macromolecular structures *in situ* (Beck and Baumeister, 2016). We vitrified cells in which ER-PM contacts were formed by tricalbins (*scs2/22*Δ *ist2*Δ cells) and subjected these cells to thinning by cryo-FIB milling and to cryo-ET (Marko et al., 2007). In resulting tomograms of cortical cell regions, we found tubular cisternae of the cER (Figures S3A and S3B). In addition, the cryo-ET data revealed densities forming bridges between the ER and PM (Figure 5A, white arrows). In some cases, the ER membrane was locally buckled toward the PM at the location of the bridging density (Figure 5A, top white arrow). Since these cells did not express VAPs and Ist2, the majority of these contacts were likely bridged by tricalbins. Occasionally, patches of PM at the contact were coated with a layer of density, reminiscent of a protein coat (Figure 5A, orange bracket).

Elevated Cytosolic Ca^{2+} Modulates Tricalbin-Mediated Contact Sites

E-Syts are regulated by Ca^{2+}, which impacts both their lipid transfer function and their capacity to bind to the PM (Bian et al., 2018; Giordano et al., 2013). We reasoned that elevated cytosolic Ca^{2+} levels might trigger functionally important structural changes that would be directly visible in *scs2/22*Δ *ist2*Δ cells by cryo-ET. To monitor cytosolic Ca^{2+} concentration, we expressed a GFP-calmodulin-fusion protein (GCaMP) as a fluorescent indicator (Carbó et al., 2017). We treated cells with 200 mM $CaCl_2$ and imaged them by live FM. Shortly after $CaCl_2$ treatment, a subpopulation of cells displayed a strong fluorescent signal, which returned to background levels within seconds, indicating transiently elevated cytosolic Ca^{2+} (Figure S3C) (Carbó et al., 2017). To target cells with high cytosolic Ca^{2+} levels for cryo-ET, we imaged cells, vitrified on EM grids, by cryo-FM prior to cryo-FIB milling (Ader et al., 2019). We thereby identified cells with strong GCaMP signals after treatment with 200 mM $CaCl_2$ (Figure 5B). In the resulting tomograms, we found ER-PM contacts similar to those in untreated cells, including cER membrane patches locally buckled toward the PM (Figure 5C, white arrows). We noticed that the PM of several ER-PM contacts was coated with a layer similar in appearance to what we observed in untreated cells (Figure 5C; Figure S3G, orange brackets). The occurrence of this layer was more frequent in cells with high Ca^{2+} (4 out of 10 tomograms) than in untreated cells (5 out of 22 tomograms). Furthermore, the layer extended over a larger fraction of the cER in cells with high Ca^{2+} (Figure 5D). To better resolve details of this layer, we performed subtomogram averaging, previously used to investigate protein coating of the PM (Bharat et al., 2018). Within each layer, we extracted subvolumes, collapsed them into 2D images, and subjected them to alignment and averaging (see STAR Methods). The resulting 2D class averages showed densities on the cytoplasmic leaflet of the PM that were on average 5.2 nm thick (SD 0.5 nm, N = 4 layers, each from a different tomogram), which were not present on PM areas outside of the contact site (Figure 5E, orange arrows and Figure S3H). Furthermore, cells with high Ca^{2+} displayed shorter intermembrane distances than untreated cells (average 21.4 nm, SD 1.9 nm, N = 21 sites in 10 cells at high Ca^{2+}; average 23.2 nm, SD 2.5 nm, N = 22 sites in 10 untreated cells) (Figure 5F). At the locations where the ER membrane was buckled toward the PM (Figure S4A), the ER-PM distances were also shorter in cells with high Ca^{2+} than in untreated cells (average 14.2 nm SD 1.4 nm, N = 19 buckles at high Ca^{2+}; average 15.2 nm, SD 1.0 nm, N = 19 buckles in untreated cells, Figure S4B).

In summary, our cryo-ET analysis indicates that tricalbin-mediated contact sites contain protein densities that bridge the two membranes. In addition, within the contact site areas, parts of the PM display a density layer reminiscent of a protein

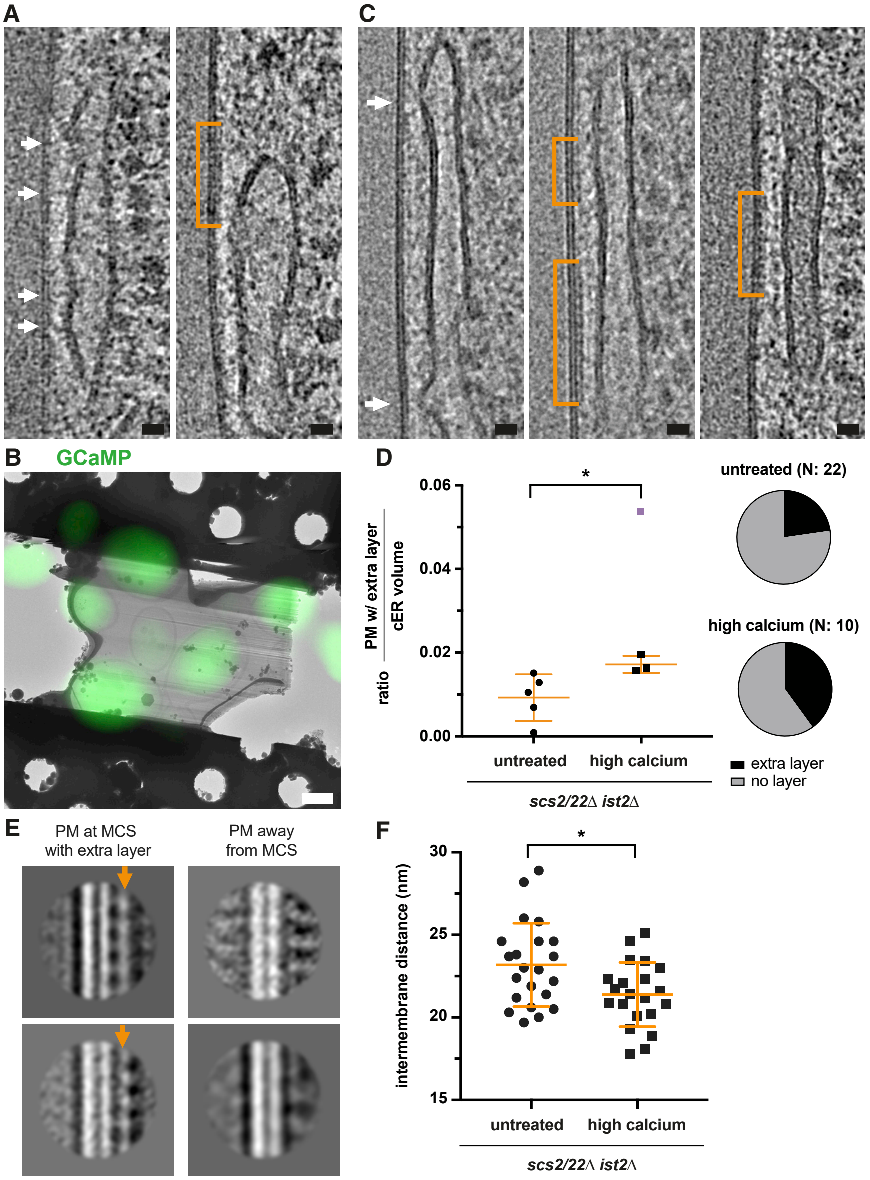

Figure 5. Protein Organization of Tricalbin-Mediated Membrane Contact Sites Depends on Ca^{2+}

(A) Virtual slices through electron cryo-tomogram of cryo-FIB milled *scs2/22Δ ist2Δ* cell. White arrows indicate densities bridging the ER and PM. Orange bracket indicates density layer on PM.

(B) Overlay of the electron cryo-microscopy (cryo-EM) image of cryo-FIB milled lamella with the cryo-FM image of GCaMP signal, acquired prior to cryo-FIB milling. Cells were treated with 200 mM $CaCl_2$ before vitrification.

(C) Virtual slices through electron cryo-tomograms of cryo-FIB milled *scs2/22Δ ist2Δ* cells, which displayed strong GCaMP signals. White arrows indicate buckling of the ER toward the PM. Orange brackets indicate density layer on the PM. The left and middle images are from the same tomogram.

(D) Ratio of PM coated with density layer to cER volume, for untreated *scs2/22Δ ist2Δ* cells and *scs2/22Δ ist2Δ* cells with strong GCaMP signals. The highest value data point (purple) in the latter data was treated as an outlier and excluded from the significance test (p = 0.0312). Pie charts indicate the fraction of electron cryo-tomograms in which extra density layers were observed.

(E) 2D class averages of PM with density layer (indicated by the orange arrow) at contact sites with the cER, compared to PM area not in contact with the cER. Extracellular PM leaflets are facing left, cytosolic leaflets right. Top and bottom averages are from the layer shown in (C) middle and right panels, respectively.

(F) Distances between ER and PM in untreated *scs2/22Δ ist2Δ* cells and in *scs2/22Δ ist2Δ* cells with strong GCaMP signals (p = 0.0126). Orange lines indicate mean and SD in (D) and (F). Scale bars: 20 nm in (A) and (C) and 2 μm in (B).

coat. This layer is more frequent and extended when cytosolic Ca^{2+} is high.

In Situ Structural Analysis of Densities Bridging Tcb3-Mediated Contact Sites

We next investigated the structure of the densities bridging the two membranes. We deleted 5 of the 6 bridging proteins (5Δ cells; *scs2/22Δ ist2Δ tcb1/2Δ*) and controlled Tcb3-GFP overexpression by a galactose-inducible promoter. We reasoned that averaging densities of which a large fraction was likely to correspond to a single tricalbin isoform would facilitate structural analysis. By live FM, we confirmed that induction by galactose was required to localize Tcb3-GFP as well as plasmid-encoded Sec63-RFP to the cell cortex in the 5Δ cells (Figure 6A). This experiment verified that overexpressed Tcb3-GFP is sufficient for formation of ER-PM contacts in 5Δ cells.

Cryo-ET of these cells revealed more extended cER regions than in the *scs2/22Δ ist2Δ* cells expressing all tricalbins at endogenous levels (Figures 6B and 6C). Occasionally, the cER membrane was buckled toward the PM. The ER-PM distance at these locations was larger than in the *scs2/22Δ ist2Δ* cells (5Δ cells: average 16.3 nm, SD = 1.2 nm, N = 10 buckles from 17 tomograms; Figure S4). Densities forming bridges between the ER and PM were distributed over all the cER (Figure 6D, white arrows). Based on the abundance of these densities in the 5Δ cells, we reasoned that a majority of them corresponded to Tcb3-GFP protein particles. While the particles were similar in appearance, their orientation relative to the plane of the PM varied. The majority of particles deviated from a perpendicular to the PM by 5°–40° (average 15.2°, SD = 12.1°, N = 1,513 particles from 17 tomograms) (Figure 6E). We extracted subvolumes containing the particles, collapsed them into 2D images, and subjected them to alignment, classification, and averaging (see STAR Methods) (Bharat and Scheres, 2016). Classification resulted in five similarly populated classes (Figure 6F). The 2D class averages all showed a similar, rod-shaped structure that connected the two membranes nearly

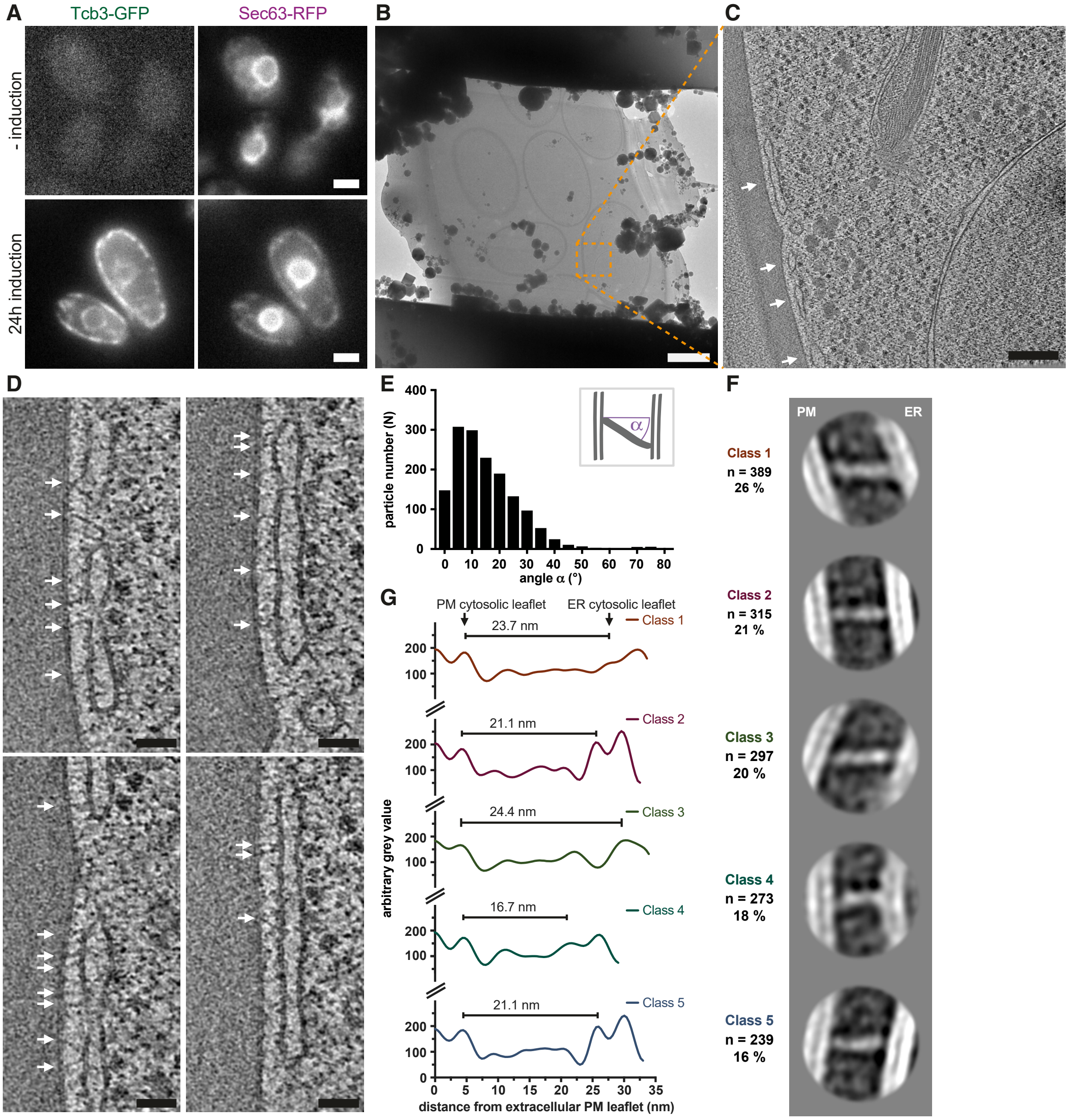

Figure 6. *In Situ* Structural Analysis of Bridging Proteins at Tcb3-Mediated Contact Sites

(A) Live FM of *scs2/22*Δ *ist2*Δ *tcb1/2*Δ *prGAL1::Tcb3::EGFP* (5Δ) cells expressing plasmid-encoded Sec63-RFP. Galactose-induced Tcb3-GFP expression rescues ER-PM contact sites, indicated by localization of Sec63-RFP to the cell cortex.

(B) Cryo-EM of cryo-FIB milled lamella through 5Δ cells after induction. Orange dashed square indicates the area corresponding to (C).

(C) Virtual slice through electron cryo-tomogram acquired at the area indicated in (B). Arrows indicate ER-PM contact sites.

(D) Magnified views of virtual slices through electron cryo-tomogram shown in (C). Arrows indicate densities bridging ER and PM.

(E) Histogram of particle orientational distribution relative to the PM; angular deviation (α°) from perpendicular, as depicted in schematic.

(F) 2D class averages of subvolumes containing bridging particles, grouped into 5 classes. Number of particles per class (n) as well as percentage of total particle set, are indicated.

(G) Projection profiles along major axes of rod-like densities in the corresponding 2D class averages. Length measurement indicated for each profile.

Scale bars: 2 μm in (A) and (B), 200 nm in (C), and 50 nm in (D).

perpendicularly (classes 2, 4, and 5) or offset by an angle of 25°–30° (classes 1 and 3). The structures displayed sequentially arranged densities (Figures 6F and S5). The density closest to the PM was tilted relative to the main axis of the particle in these classes. While densities corresponding to the two leaflets of the PM were similar in all classes, the cER membrane appeared ruffled to various degrees and displayed a curvature of 34 nm radius in class 4. We estimated the length of the structures by measuring the distance between the cytosolic leaflets of PM and ER using line profiles projected along the structure axes

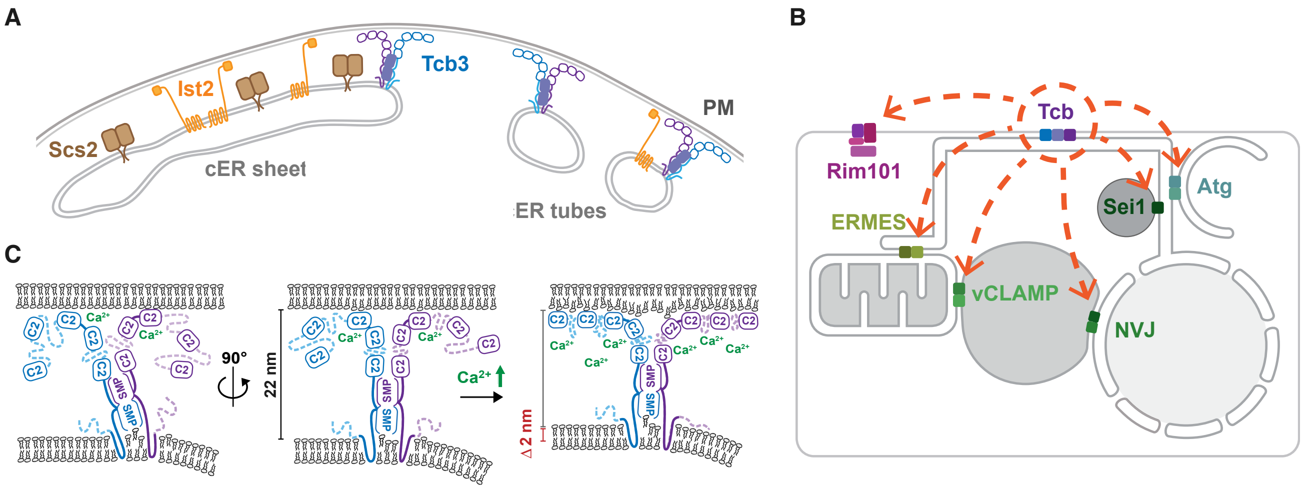

Figure 7. Model for the Functionality of Tricalbins on the Ultrastructural, Cellular, and Molecular Level

(A) The distribution of ER-PM proteins correlates with cER membrane curvature but not with intermembrane distance. Tricalbins preferably localize to high-curvature cER.

(B) Selected genetic interactions between tricalbins and components of lipid pathways highlight redundancies in cellular lipid fluxes at organelle contact sites and suggest that tricalbins are implicated in the maintenance of PM lipid asymmetry.

(C) Tcb3-mediated contacts contain rod-like bridges between the two membranes, presumably tricalbin structures. The rod is tilted relative to the PM plane and may appear perpendicular at a rotated viewing angle. It bridges an intermembrane distance of ~22 nm. This organization implies that the dimeric SMP tube arranges along the rod axis. The Tcb3 membrane domain mediates curvature preference, possibly facilitating lipid extraction and/or insertion. Probably not all C2 domains are bound to the PM. Upon increased Ca^{2+} concentrations the intermembrane distance shortens by 2 nm. This could either cause, or be caused by, binding of more C2 domains to the PM. The C2 domains could form a dense membrane coat and potentially introduce bilayer disorder.

(Figure 6G). In four of the five classes, the structure lengths were 21–24 nm, whereas in class 4 the length was 16.7 nm. Thus, the majority of the particles were very similar in size and structure and differed mainly in their orientation relative to the membranes. A small fraction of particles (class 4) were shorter and associated with higher cER curvature.

In summary, our analysis shows that contact sites induced by Tcb3 overexpression contain rod-shaped structures that bridge the intermembrane space through linearly arranged densities. These structures are tilted relative to the PM plane and have characteristic lengths.

DISCUSSION

ER-PM Contact Sites Are Organized into Compositionally and Ultrastructurally Different Regions

Mammalian ER-PM contacts mediated by E-Syts appear ultrastructurally distinct from those driving store-operated Ca^{2+} entry, mediated by STIM1, suggesting a functional aspect to MCS architecture (Fernández-Busnadiego et al., 2015). Yet, it remains unclear to what extent different proteins segregate within MCSs and what the mechanism driving segregation might be. We show here that despite a large degree of coincident localization along the cER, different bridging proteins are not distributed homogenously, suggesting that ER-PM contact sites are organized into subdomains of varied composition.

Exclusion of specific proteins could be based on size and driven by intermembrane distances (Hoffmann and Kukulski, 2017), as described for cell-cell interactions in T cell responses and between giant unilamellar vesicles *in vitro* (James and Vale, 2012; Schmid et al., 2016). Distances between the ER and PM are known to be highly variable (West et al., 2011), and it has been hypothesized that different bridging proteins could mediate different distances (Gatta and Levine, 2016; West et al., 2011). However, we find that all bridging proteins associated with very similar average intermembrane distances. The bridging proteins are thus not the major regulators of intermembrane distance, although they might set constraints to the observed variability. As this variability was similarly large for all bridging proteins, segregation is unlikely to be driven by size-based exclusion.

Instead, our results and those of (Collado et al., 2019) reveal that the localization of different bridging proteins correlates with differences in cER curvature. In wild-type cells each bridging protein family had a distinct preference for a particular membrane curvature; VAPs and Ist2 localized to cER sheets, whereas tricalbins localized to the tubular cER and curved edges of cER sheets. We suggest that local cER membrane curvature, rather than intermembrane distance, determines protein organization at ER-PM contact sites (Figure 7A). Localization to cER domains of specific curvature could ensure that the bridging proteins are in proximity to functional partners with similar curvature preference.

Curvature Sensitivity of Tricalbins within the cER

We found that the preference of tricalbins for tubular cER depends on its membrane domain, predicted to form a hairpin-like structure in the ER membrane similar to the curvature-generating reticulons (Giordano et al., 2013; Voeltz et al., 2006). In wild-type cells Tcb3 overlapped extensively with the pan-ER

translocon component Sec63, whereas in the absence of the major reticulons, Tcb3 and Sec63 localized to separate cER pools: sheet-like cER that contained Sec63 and highly tubular Tcb3-positive membranes. These findings indicate that reticulons might modulate overall cER curvature to jointly accommodate curvature-preferring proteins such as tricalbins and bulkier components such as the translocon. Without this modulation, domains of high residual curvature exclude the translocon, while tricalbins enrich there or may even generate this curvature themselves. Previous work showed that high membrane curvature facilitates lipid transfer *in vitro*, likely due to bilayer packing defects that promote extraction of lipid molecules (Moser von Filseck et al., 2015). Given the lipid transfer function of E-Syts (Saheki et al., 2016; Yu et al., 2016), we speculate that the curvature sensitivity of tricalbins could facilitate lipid transfer between the ER and PM.

Tricalbin Function Overlaps with Pathways for Cellular Distribution of Lipids

Tricalbins and E-Syts are not essential for cell fitness (Manford et al., 2012; Saheki et al., 2016; Toulmay and Prinz, 2012) or mouse physiology (Sclip et al., 2016; Tremblay and Moss, 2016). Even in yeast, the lack of phenotype upon deletion has made it difficult to assess their cellular function (Manford et al., 2012; Omnus et al., 2016). We hypothesized that compensatory pathways or redundant protein functions mask phenotypes. Systematic screens using yeast deletion libraries can reveal genetic interactions that provide hints at functional relationships (Costanzo et al., 2016). This technology allowed us to uncover cellular pathways that specifically overlap with tricalbins and to assess the importance of the curvature-sensitive domain, the putative lipid transfer domain and the C2 domains. We found synthetic growth phenotypes associated with simultaneous loss of all three tricalbins and other MCS components, such as components of the nucleus-vacuole junction, supporting the model that MCSs function as a cross-regulated network (Lang et al., 2015) (Figure 7B). Through LTPs that localize to distinct MCSs, the intracellular routes of lipid molecules are thought to be redundant (Kumar et al., 2018; Lang et al., 2015). Another potential role for tricalbins in lipid flow is revealed by the autophagy-related hits. Atg2 acts as LTP and the tether between the pre-autophagosomal structure and the ER, promoting expansion of the isolation membrane (Kotani et al., 2018; Maeda et al., 2019; Osawa et al., 2019; Valverde et al., 2019). E-Syts have also been proposed to contribute to autophagosome formation (Nascimbeni et al., 2017). Our finding that autophagy genes, especially Atg2, genetically interact with tricalbins suggests a model in which lipid transfer for autophagosome formation could involve multiple, redundant routes.

Of particular interest is the previously unknown potential role for tricalbins in PM lipid asymmetry, suggested by genetic interactions with RIM101 pathway components (Figure 7B). This pathway senses and induces adaptation to changes in pH and in the asymmetric distribution of lipids across the PM bilayer, possibly by the Rim21 sensory complex detecting changes in PM surface charge (Obara et al., 2012; Rockenfeller et al., 2018). A transcriptional response ultimately induces expression of PM floppases that re-establish lipid asymmetry in the PM (Ikeda et al., 2008). Although spatially segregated from ER-PM contact sites, the Rim101 pathway is constitutively active in yeast cells lacking all cER (Obara and Kihara, 2017). Our data indicate that this activity is likely due to the loss of tricalbins, rather than lack of the cER, as our query strains retain ER-PM contacts mediated by Ist2 and VAPs. We speculate that tricalbins contribute to PM asymmetry, possibly by lipid transfer, in the absence of which lipid asymmetry is restored through the Rim101 rescue program. The distribution of lipids across the two bilayer leaflets is fundamental to multiple aspects of membrane function, including signaling, membrane potential, and curvature generation (Gurtovenko and Vattulainen, 2008; McMahon and Boucrot, 2015; Segawa and Nagata, 2015). Precisely which lipids are affected by the loss of tricalbin function remains to be determined.

The Major Protein Domains of Tcb3 Are Functionally Dispensable

Surprisingly, Tcb3 function could largely be rescued by mutants in which we replaced its curvature sensitivity, its putative lipid transfer domain, or its set of C2 domains. We consider that either the molecular functions of these domains are not relevant for cellular roles of Tcb3 or these domains perform redundant functions. Indeed, reticulons drive ER curvature and facilitate lipid transfer (Voss et al., 2012) and thus might compensate when Tcb3 loses its curvature-sensitive properties. Similarly, lipid transfer via the SMP domain (Saheki et al., 2016) may be replaced by other LTPs with lipid-binding domains, such as Vps13 (Kumar et al., 2018). The C2 domains might provide a way of regulating PM binding by Ca^{2+} (Bian et al., 2018), but regulation might only be required under certain circumstances. The robust rescues by various Tcb3 constructs indicate that the functionality of tricalbins not only overlaps with cellular pathways but possibly also with individual protein domains.

The Protein Organization of Tricalbin-Mediated Contact Sites Is Ca^{2+} Dependent

Ca^{2+} stimulation had marked effects on tricalbin-mediated contacts, resulting in increased density on the PM, reminiscent of a protein coat. The thickness of this putative coat is compatible with a layer of densely packed C2 domains. Previous biochemical data suggested that Ca^{2+} binding displaces an E-Syt1 C2 domain from an occluding position on the SMP channel, relocating the C2 domain to the PM (Bian et al., 2018). Many C2 domains bind PIP_2 in a Ca^{2+}-dependent manner, thereby regulating interaction with target membranes (Schiavo et al., 1996). Our *in situ* data do not rule out that the layer could be formed by other proteins that bind the PM in a Ca^{2+} responsive manner. However, our data are consistent with a model in which Ca^{2+} enhances binding of tricalbin C2 domains to the PM. Interactions of C2 domains with lipids can change bilayer structure and induce membrane curvature (Martens et al., 2007). Thus, engagement of the tricalbin C2 domains with the PM could facilitate insertion or extraction of lipid molecules by inducing disorder in the bilayer (Figure 7C). A second effect of high Ca^{2+} was a shortening of the distance between the ER and PM from 23 to 21 nm. This is a more modest change than has been observed in mammalian cells upon thapsigargin treatment, which induced a narrowing of E-Syt1-mediated contacts from 22 to 15 nm (Fernández-Busnadiego et al., 2015). Based on our results, we speculate that

under normal Ca^{2+} conditions, not all C2 domains of the tricalbins are bound to the PM. At high Ca^{2+}, coincident with reduced ER-PM distance, more C2 domains might bind to the PM and at maximal binding could form a densely packed coat (Figure 7C).

Rod-Shaped Structures at Tcb3-Mediated Contacts Suggest an Intermembrane Architecture for Tricalbins

Subtomogram averaging permits an *in situ* structural analysis of the major protein densities present at contacts mediated by Tcb3 alone. Given the abundance of Tcb3 at these contacts, averages of these densities likely revealed architectural features of Tcb3. This architecture consists of remarkably rigid rods that are mostly oriented nearly perpendicular to the PM plane. Different 2D class averages shared significant similarities, suggesting that they mostly represent different views of a single structure. This would imply that the structure is tilted by approximately 20° at its highest inclination, appearing perpendicular at views rotated by 90° round the membrane normal (Figure 7C). The lengths of the average particles in the respective classes are consistent with this model. Because of the difficulty to accurately align the rod-shaped particles in three dimensions using inherently noisy cellular cryo-ET data, we were unable to obtain a reliable 3D reconstruction.

In wild-type cells, the architecture of tricalbins and membranes may be different. E-Syts were described to homo- and heterodimerize (Giordano et al., 2013). In the 5Δ cells, Tcb3 can only form homodimers. Heterodimerization could induce different structural arrangements of tricalbins. Furthermore, in wild-type cells, the presence of Scs2/22 and Ist2 could influence membrane composition and the organization of tricalbins.

Multiple structural studies showed that SMP domains dimerize into head-to-head barrels, forming a 3 nm wide and 9 nm long tube in the case of E-Syt2 (AhYoung et al., 2015; Kawano et al., 2018; Schauder et al., 2014). The central densities in our 2D class averages are compatible with an SMP dimer oriented along the particle axis (Figure 7C). This arrangement would exclude a model in which the SMP dimer forms a shuttle parallel to the membrane. This model has been favored because a central density parallel to the membranes was observed in E-Syt1 overexpressing cells and was enhanced upon Ca^{2+} stimulation (Fernández-Busnadiego et al., 2015; Reinisch and De Camilli, 2016). Although we also detect extra density at tricalbin-mediated contacts in the presence of high Ca^{2+}, the layer we describe lies immediately adjacent to the PM. This coat-like layer is similar to PM-bound BAR domains (Bharat et al., 2018) and is suggestive of an intimate interaction with the PM, more likely to be driven by Ca^{2+}-responsive C2 domains than by SMP domains.

An open question about lipid transfer at MCS is the mode of lipid travel across the soluble interface between bilayers. One model is direct tunneling of lipid molecules from one to the other bilayer through the 9 nm SMP dimer barrel, which requires intermembrane distances in the range of 10 nm (Schauder et al., 2014). We measured the shortest distances at positions of local cER buckling toward the PM to be ~14 nm, which was similar to the length of the shortest structure in 2D class averages. Over larger contact areas, the measured distances of 21–24 nm corresponded to the length of most of the particles. Thus, under all circumstances we analyzed, the distance across which lipid molecules must be transferred was too large to be compatible with tunneling via SMP dimers alone. This could mean that tunneling is a transient event that we fail to capture in our analysis. Alternatively, an additional transfer mechanism could bridge the remaining 5–12 nm. A third possibility is that tricalbins, unlike mammalian E-Syts, do not transfer lipids via their SMP domains, but contribute to lipid distribution in an indirect way, for instance through unknown binding partners.

Conclusions

The unprecedented *in situ* structural view on proteins bridging two organelles provides an essential basis for addressing the molecular mechanism of lipid transfer. Our CLEM results reveal how the specific ultrastructure of ER-PM contacts depends on the bridging proteins, implying that contact site function is regulated through variability in composition as well as architecture, potentially adjusted to their cellular roles. We suggest such a possible involvement for tricalbins in maintenance of bilayer asymmetry in the PM. Our study shows a way to untangle the intricate network of redundancies at the protein domain and pathway level, which emerge as a fundamental principle of the cell's overlapping lipid distribution routes.

STAR★METHODS

Detailed methods are provided in the online version of this paper and include the following:

- KEY RESOURCES TABLE
- LEAD CONTACT AND MATERIALS AVAILABILITY
- EXPERIMENTAL MODEL AND SUBJECT DETAILS
  - Yeast Strains
- METHOD DETAILS
  - Yeast Genetic Techniques
  - Plasmid Constructs and Cloning
  - Live-cell Imaging
  - SGA Analysis
  - Western Blot
  - Room-temperature Correlative Light and Electron Microscopy
  - Classification and Quantitative analysis of ER-PM Contacts Identified by RT-CLEM
  - Cryo-EM Sample Preparation
  - Cryo-FIB Milling
  - Cryo-fluorescence Microscopy
  - Electron Cryo-tomography
  - Image Processing and Classification of Cryo-ET Data
- QUANTIFICATION AND STATISTICAL ANALYSIS
- DATA AND CODE AVAILABILITY

SUPPLEMENTAL INFORMATION

Supplemental Information can be found online at https://doi.org/10.1016/j.devcel.2019.09.019.

ACKNOWLEDGMENTS

We thank the Electron Microscopy and Light Microscopy Facilities of the MRC-LMB for support in data collection, Jake Grimmett and Toby Darling for computing support, Julia Mahamid for instructions on cryo-FIB milling, and Christopher Russo for help in using the Scios microscope. We thank

Ruben Fernandez-Busnadiego and Javier Collado for sharing unpublished results. We thank Pablo S. Aguilar for the kind gift of the GCaMP plasmid, Susan Michaelis for the Sec63-RFP plasmid, and Wendell Lim and Kurt Thorn for the mRuby2 plasmid. This work was supported by the Medical Research Council (MC_UP_1201/8 to W.K. and MC_UP_1201/10 to E.A.M.). T.A.M.B. is funded by a Sir Henry Dale Fellowship from the Wellcome Trust and the Royal Society (202231/Z/16/Z) and a Vallee Foundation Scholarship.

AUTHOR CONTRIBUTIONS

Conceptualization, P.C.H., E.A.M., and W.K.; Methodology, P.C.H., T.A.M.B., and J.B.; Formal Analysis, P.C.H., T.A.M.B., J.B., and W.K.; Investigation, P.C.H., T.A.M.B., M.R.W., E.A.M., and W.K.; Writing – Original Draft, P.C.H. and W.K.; Writing – Review and Editing, P.C.H., T.A.M.B., M.R.W., E.A.M., and W.K.

DECLARATION OF INTERESTS

The authors declare no competing interests.

Received: March 28, 2019
Revised: August 6, 2019
Accepted: September 25, 2019
Published: November 18, 2019

REFERENCES

Ader, N.R., Hoffmann, P.C., Ganeva, I., Borgeaud, A.C., Wang, C., Youle, R.J., and Kukulski, W. (2019). Molecular and topological reorganizations in mitochondrial architecture interplay during Bax-mediated steps of apoptosis. Elife *8*, e40712.

Ader, N.R., and Kukulski, W. (2017). triCLEM: combining high-precision, room temperature CLEM with cryo-fluorescence microscopy to identify very rare events. Methods Cell Biol. *140*, 303–320.

AhYoung, A.P., Jiang, J., Zhang, J., Khoi Dang, X., Loo, J.A., Zhou, Z.H., and Egea, P.F. (2015). Conserved SMP domains of the ERMES complex bind phospholipids and mediate tether assembly. Proc. Natl. Acad. Sci. USA *112*, E3179–E3188.

Beck, M., and Baumeister, W. (2016). Cryo-electron tomography: can it reveal the molecular sociology of cells in atomic detail? Trends Cell Biol. *26*, 825–837.

Bharat, T.A.M., Hoffmann, P.C., and Kukulski, W. (2018). Correlative microscopy of vitreous sections provides insights into BAR-Domain Organization in situ. Structure *26*, 879–886.e3.

Bharat, T.A.M., Russo, C.J., Löwe, J., Passmore, L.A., and Scheres, S.H.W. (2015). Advances in single-particle electron cryomicroscopy structure determination applied to sub-tomogram averaging. Structure *23*, 1743–1753.

Bharat, T.A.M., and Scheres, S.H.W. (2016). Resolving macromolecular structures from electron cryo-tomography data using subtomogram averaging in RELION. Nat. Protoc *11*, 2054–2065.

Bian, X., Saheki, Y., and De Camilli, P. (2018). Ca^{2+} releases E-Syt1 autoinhibition to couple ER-plasma membrane tethering with lipid transport. EMBO J *37*, 219–234.

Brunner, J.D., Lim, N.K., Schenck, S., Duerst, A., and Dutzler, R. (2014). X-ray structure of a calcium-activated TMEM16 lipid scramblase. Nature *516*, 207–212.

Carbó, N., Tarkowski, N., Ipiña, E.P., Dawson, S.P., and Aguilar, P.S. (2017). Sexual pheromone modulates the frequency of cytosolic Ca^{2+} bursts in Saccharomyces cerevisiae. Mol. Biol. Cell *28*, 501–510.

Costanzo, M., VanderSluis, B., Koch, E.N., Baryshnikova, A., Pons, C., Tan, G., Wang, W., Usaj, M., Hanchard, J., Lee, S.D., et al. (2016). A global genetic interaction network maps a wiring diagram of cellular function. Science *353*, aaf1420.

Creutz, C.E., Snyder, S.L., and Schulz, T.A. (2004). Characterization of the yeast tricalbins: membrane-bound multi-C2-domain proteins that form complexes involved in membrane trafficking. Cell. Mol. Life Sci. *61*, 1208–1220.

Collado, J., Kalemanov, M., Campelo, F., Bourgoint, C., Thomas, F., Loewith, R., Martínez-Sánchez, A., Baumeister, W., Stefan, C.J., and Fernández-Busnadiego, R. (2019). Tricalbin-Mediated Contact Sites Control ER Curvature to Maintain Plasma Membrane Integrity. Dev. Cell *51*. Published online November 18, 2019. https://doi.org/10.1016/j.devcel.2019.10.018.

de Chaumont, F., Dallongeville, S., Chenouard, N., Hervé, N., Pop, S., Provoost, T., Meas-Yedid, V., Pankajakshan, P., Lecomte, T., Le Montagner, Y., et al. (2012). Icy: an open BioImage informatics platform for extended reproducible research. Nat. Methods *9*, 690–696.

Delneri, D., Gardner, D.C., Bruschi, C.V., and Oliver, S.G. (1999). Disruption of seven hypothetical aryl alcohol dehydrogenase genes from *Saccharomyces cerevisiae* and construction of a multiple knock-out strain. Yeast *15*, 1681–1689.

Fernández-Busnadiego, R., Saheki, Y., and De Camilli, P. (2015). Three-dimensional architecture of extended synaptotagmin-mediated endoplasmic reticulum-plasma membrane contact sites. Proc. Natl. Acad. Sci. USA *112*, E2004–E2013.

Frangakis, A.S., and Hegerl, R. (2001). Noise reduction in electron tomographic reconstructions using nonlinear anisotropic diffusion. J. Struct. Biol. *135*, 239–250.

Gatta, A.T., and Levine, T.P. (2016). Piecing together the patchwork of contact sites. Trends Cell Biol. *27*, 1–16.

Gatta, A.T., Wong, L.H., Sere, Y.Y., Calderón-Noreña, D.M., Cockcroft, S., Menon, A.K., and Levine, T.P. (2015). A new family of StART domain proteins at membrane contact sites has a role in ER-PM sterol transport. Elife *4*, e07253.

Giordano, F., Saheki, Y., Idevall-Hagren, O., Colombo, S.F., Pirruccello, M., Milosevic, I., Gracheva, E.O., Bagriantsev, S.N., Borgese, N., and De Camilli, P. (2013). PI(4,5)P_2-dependent and Ca^{2+}-regulated ER-PM interactions mediated by the extended synaptotagmins. Cell *153*, 1494–1509.

Gurtovenko, A.A., and Vattulainen, I. (2008). Membrane potential and electrostatics of phospholipid bilayers with asymmetric transmembrane distribution of anionic lipids. J. Phys. Chem. B *112*, 4629–4634.

Hagen, W.J.H., Wan, W., and Briggs, J.A.G. (2017). Implementation of a cryo-electron tomography tilt-scheme optimized for high resolution subtomogram averaging. J. Struct. Biol. *197*, 191–198.

Hayashi, M., Fukuzawa, T., Sorimachi, H., and Maeda, T. (2005). Constitutive activation of the pH-responsive Rim101 pathway in yeast mutants defective in late steps of the MVB/ESCRT pathway. Mol. Cell. Biol. *25*, 9478–9490.

Hoffmann, P.C., and Kukulski, W. (2017). Perspective on architecture and assembly of membrane contact sites. Biol. Cell *109*, 400–408.

Hohmann-Marriott, M.F., Sousa, A.A., Azari, A.A., Glushakova, S., Zhang, G., Zimmerberg, J., and Leapman, R.D. (2009). Nanoscale 3D cellular imaging by axial scanning transmission electron tomography. Nat. Methods *6*, 729–731.

Hu, J., Shibata, Y., Voss, C., Shemesh, T., Li, Z., Coughlin, M., Kozlov, M.M., Rapoport, T.A., and Prinz, W.A. (2008). Membrane proteins of the endoplasmic reticulum induce high-curvature tubules. Science *319*, 1247–1250.

Ikeda, M., Kihara, A., Denpoh, A., and Igarashi, Y. (2008). The Rim101 pathway is involved in Rsb1 expression induced by altered lipid asymmetry. Mol. Biol. Cell *19*, 1922–1931.

James, J.R., and Vale, R.D. (2012). Biophysical mechanism of T-cell receptor triggering in a reconstituted system. Nature *487*, 64–69.

Janke, C., Magiera, M.M., Rathfelder, N., Taxis, C., Reber, S., Maekawa, H., Moreno-Borchart, A., Doenges, G., Schwob, E., Schiebel, E., et al. (2004). A versatile toolbox for PCR-based tagging of yeast genes: new fluorescent proteins, more markers and promoter substitution cassettes. Yeast *21*, 947–962.

Kawano, S., Tamura, Y., Kojima, R., Bala, S., Asai, E., Michel, A.H., Kornmann, B., Riezman, I., Riezman, H., Sakae, Y., et al. (2018). Structure-function insights into direct lipid transfer between membranes by Mmm1-Mdm12 of ERMES. J. Cell Biol. *217*, 959–974.

Khmelinskii, A., Meurer, M., Duishoev, N., Delhomme, N., and Knop, M. (2011). Seamless gene tagging by endonuclease-driven homologous recombination. PLOS One *6*, e23794.

Kopec, K.O., Alva, V., and Lupas, A.N. (2010). Homology of SMP domains to the TULIP superfamily of lipid-binding proteins provides a structural basis for lipid exchange between ER and mitochondria. Bioinformatics *26*, 1927–1931.

Kornmann, B., Currie, E., Collins, S.R., Schuldiner, M., Nunnari, J., Weissman, J.S., and Walter, P. (2009). An ER-mitochondria tethering complex revealed by a synthetic biology screen. Science *325*, 477–481.

Kotani, T., Kirisako, H., Koizumi, M., Ohsumi, Y., and Nakatogawa, H. (2018). The Atg2-Atg18 complex tethers pre-autophagosomal membranes to the endoplasmic reticulum for autophagosome formation. Proc. Natl. Acad. Sci. USA *115*, 10363–10368.

Kremer, J.R., Mastronarde, D.N., and McIntosh, J.R. (1996). Computer visualization of three-dimensional image data using IMOD. J. Struct. Biol. *116*, 71–76.

Kukulski, W., Schorb, M., Welsch, S., Picco, A., Kaksonen, M., and Briggs, J.A.G. (2011). Correlated fluorescence and 3D electron microscopy with high sensitivity and spatial precision. J. Cell Biol. *192*, 111–119.

Kumar, N., Leonzino, M., Hancock-Cerutti, W., Horenkamp, F.A., Li, P., Lees, J.A., Wheeler, H., Reinisch, K.M., and De Camilli, P. (2018). VPS13A and VPS13C are lipid transport proteins differentially localized at ER contact sites. J. Cell Biol. *217*, 3625–3639.

Lamb, T.M., Xu, W., Diamond, A., and Mitchell, A.P. (2001). Alkaline response genes of Saccharomyces cerevisiae and their relationship to the RIM101 pathway. J. Biol. Chem. *276*, 1850–1856.

Lang, A.B., John Peter, A.T., Walter, P., and Kornmann, B. (2015). ER-mitochondrial junctions can be bypassed by dominant mutations in the endosomal protein Vps13. J. Cell Biol. *210*, 883–890.

Lee, S., Lim, W.A., and Thorn, K.S. (2013). Improved blue, green, and red fluorescent protein tagging vectors for S. cerevisiae. PLoS One *8*, e67902.

Loewen, C.J.R., and Levine, T.P. (2005). A highly conserved binding site in vesicle-associated membrane protein-associated protein (VAP) for the FFAT motif of lipid-binding proteins. J. Biol. Chem. *280*, 14097–14104.

Loewen, C.J.R., Roy, A., and Levine, T.P. (2003). A conserved ER targeting motif in three families of lipid binding proteins and in Opi1p binds VAP. EMBO J *22*, 2025–2035.

Loewen, C.J.R., Young, B.P., Tavassoli, S., and Levine, T.P. (2007). Inheritance of cortical ER in yeast is required for normal septin organization. J. Cell Biol. *179*, 467–483.

Longtine, M.S., McKenzie, A., 3rd, Demarini, D.J., Shah, N.G., Wach, A., Brachat, A., Philippsen, P., and Pringle, J.R. (1998). Additional modules for versatile and economical PCR-based gene deletion and modification in Saccharomyces cerevisiae. Yeast *14*, 953–961.

Maeda, S., Otomo, C., and Otomo, T. (2019). The autophagic membrane tether ATG2A transfers lipids between membranes. Elife *8*, e45777.

Manford, A.G., Stefan, C.J., Yuan, H.L., MacGurn, J.A., and Emr, S.D. (2012). ER-to-plasma membrane tethering proteins regulate cell signaling and ER morphology. Dev. Cell *23*, 1129–1140.

Marko, M., Hsieh, C., Schalek, R., Frank, J., and Mannella, C. (2007). Focused-ion-beam thinning of frozen-hydrated biological specimens for cryo-electron microscopy. Nat. Methods *4*, 215–217.

Martens, S., Kozlov, M.M., and McMahon, H.T. (2007). How synaptotagmin promotes membrane fusion. Science *316*, 1205–1208.

Mastronarde, D.N. (1997). Dual-axis tomography: an approach with alignment methods that preserve resolution. J. Struct. Biol. *120*, 343–352.

Mastronarde, D.N. (2005). Automated electron microscope tomography using robust prediction of specimen movements. J. Struct. Biol. *152*, 36–51.

McMahon, H.T., and Boucrot, E. (2015). Membrane curvature at a glance. J. Cell Sci. *128*, 1065–1070.

Metzger, M.B., Maurer, M.J., Dancy, B.M., and Michaelis, S. (2008). Degradation of a cytosolic protein requires endoplasmic reticulum-associated degradation machinery. J. Biol. Chem. *283*, 32302–32316.

Monk, B.C., Tomasiak, T.M., Keniya, M.V., Huschmann, F.U., Tyndall, J.D.A., O'Connell, J.D., Cannon, R.D., McDonald, J.G., Rodriguez, A., Finer-Moore, J.S., et al. (2014). Architecture of a single membrane spanning cytochrome P450 suggests constraints that orient the catalytic domain relative to a bilayer. Proc. Natl. Acad. Sci. USA *111*, 3865–3870.

Moser von Filseck, J., Vanni, S., Mesmin, B., Antonny, B., and Drin, G. (2015). A phosphatidylinositol-4-phosphate powered exchange mechanism to create a lipid gradient between membranes. Nat. Commun *6*, 6671.

Murphy, S.E., and Levine, T.P. (2016). VAP, a Versatile Access Point for the endoplasmic reticulum: review and analysis of FFAT-like motifs in the VAPome. Biochim. Biophys. Acta *1861*, 952–961.

Nascimbeni, A.C., Giordano, F., Dupont, N., Grasso, D., Vaccaro, M.I., Codogno, P., and Morel, E. (2017). ER–plasma membrane contact sites contribute to autophagosome biogenesis by regulation of local PI3P synthesis. EMBO J. *36*, 2018–2033.

Nixon-Abell, J., Obara, C.J., Weigel, A.V., Li, D., Legant, W.R., Xu, C.S., Pasolli, H.A., Harvey, K., Hess, H.F., Betzig, E., et al. (2016). Increased spatiotemporal resolution reveals highly dynamic dense tubular matrices in the peripheral ER. Science *354*, aaf3928.

Obara, K., and Kihara, A. (2017). The Rim101 pathway contributes to ER stress adaptation through sensing the state of plasma membrane. Biochem. J. *474*, 51–63.

Obara, K., Yamamoto, H., and Kihara, A. (2012). Membrane protein Rim21 plays a central role in sensing ambient pH in Saccharomyces cerevisiae. J. Biol. Chem. *287*, 38473–38481.

Omnus, D.J., Manford, A.G., Bader, J.M., Emr, S.D., and Stefan, C.J. (2016). Phosphoinositide kinase signaling controls ER-PM cross-talk. Mol. Biol. Cell *27*, 1170–1180.

Osawa, T., Kotani, T., Kawaoka, T., Hirata, E., Suzuki, K., Nakatogawa, H., Ohsumi, Y., and Noda, N.N. (2019). Atg2 mediates direct lipid transfer between membranes for autophagosome formation. Nat. Struct. Mol. Biol. *26*, 281–288.

Pan, X., Roberts, P., Chen, Y., Kvam, E., Shulga, N., Huang, K., Lemmon, S., and Goldfarb, D.S. (2000). Nucleus-vacuole junctions in Saccharomyces cerevisiae are formed through the direct interaction of Vac8p with Nvj1p. Mol. Biol. Cell *11*, 2445–2457.

Paul-Gilloteaux, P., Heiligenstein, X., Belle, M., Domart, M.C., Larijani, B., Collinson, L., Raposo, G., and Salamero, J. (2017). eC-CLEM: flexible multidimensional registration software for correlative microscopies. Nat. Methods *14*, 102–103.

Quon, E., Sere, Y.Y., Chauhan, N., Johansen, J., Sullivan, D.P., Dittman, J.S., Rice, W.J., Chan, R.B., Di Paolo, G., Beh, C.T., et al. (2018). Endoplasmic reticulum-plasma membrane contact sites integrate sterol and phospholipid regulation. PLoS Biol. *16*, e2003864.

Reid, R.J.D., González-Barrera, S., Sunjevaric, I., Alvaro, D., Ciccone, S., Wagner, M., and Rothstein, R. (2011). Selective ploidy ablation, a high-throughput plasmid transfer protocol, identifies new genes affecting topoisomerase I-induced DNA damage. Genome Res. *21*, 477–486.

Reinisch, K.M., and De Camilli, P. (2016). SMP-domain proteins at membrane contact sites: structure and function. Biochim. Biophys. Acta *1861*, 924–927.

Rockenfeller, P., Smolnig, M., Diessl, J., Bashir, M., Schmiedhofer, V., Knittelfelder, O., Ring, J., Franz, J., Foessl, I., Khan, M.J., et al. (2018). Diacylglycerol triggers Rim101 pathway-dependent necrosis in yeast: a model for lipotoxicity. Cell Death Differ *25*, 767–783.

Russo, C.J., Scotcher, S., and Kyte, M. (2016). A precision cryostat design for manual and semi-automated cryo-plunge instruments. Rev. Sci. Instrum *87*, 114302.

Saheki, Y., Bian, X., Schauder, C.M., Sawaki, Y., Surma, M.A., Klose, C., Pincet, F., Reinisch, K.M., and De Camilli, P. (2016). Control of plasma membrane lipid homeostasis by the extended synaptotagmins. Nat. Cell Biol. *18*, 504–515.

Saheki, Y., and De Camilli, P. (2017). Endoplasmic reticulum-plasma membrane contact sites. Annu. Rev. Biochem *86*, 659–684.

Schaffer, M., Engel, B.D., Laugks, T., Mahamid, J., Plitzko, J.M., and Baumeister, W. (2015). Cryo-focused ion beam sample preparation for imaging vitreous cells by cryo-electron tomography. Bio Protoc. *5*, e1575.

Schaffer, M., Mahamid, J., Engel, B.D., Laugks, T., Baumeister, W., and Plitzko, J.M. (2017). Optimized cryo-focused ion beam sample preparation aimed at in situ structural studies of membrane proteins. J. Struct. Biol. *197*, 73–82.

Schauder, C.M., Wu, X., Saheki, Y., Narayanaswamy, P., Torta, F., Wenk, M.R., De Camilli, P., and Reinisch, K.M. (2014). Structure of a lipid-bound extended synaptotagmin indicates a role in lipid transfer. Nature *510*, 552–555.

Scheres, S.H.W. (2012). RELION: implementation of a Bayesian approach to cryo-EM structure determination. J. Struct. Biol. *180*, 519–530.

Schiavo, G., Gu, Q.M., Prestwich, G.D., Söllner, T.H., and Rothman, J.E. (1996). Calcium-dependent switching of the specificity of phosphoinositide binding to synaptotagmin. Proc. Natl. Acad. Sci. USA *93*, 13327–13332.

Schindelin, J., Arganda-Carreras, I., Frise, E., Kaynig, V., Longair, M., Pietzsch, T., Preibisch, S., Rueden, C., Saalfeld, S., Schmid, B., et al. (2012). Fiji: an open-source platform for biological-image analysis. Nat. Methods *9*, 676–682.

Schmid, E.M., Bakalar, M.H., Choudhuri, K., Weichsel, J., Ann, H.S., Geissler, P.L., Dustin, M.L., and Fletcher, D.A. (2016). Size-dependent protein segregation at membrane interfaces. Nat. Phys. *12*, 704–711.

Schorb, M., Gaechter, L., Avinoam, O., Sieckmann, F., Clarke, M., Bebeacua, C., Bykov, Y.S., Sonnen, A.F., Lihl, R., and Briggs, J.A.G. (2017). New hardware and workflows for semi-automated correlative cryo-fluorescence and cryo-electron microscopy/tomography. J. Struct. Biol. *197*, 83–93.

Schuldiner, M., Collins, S.R., Thompson, N.J., Denic, V., Bhamidipati, A., Punna, T., Ihmels, J., Andrews, B., Boone, C., Greenblatt, J.F., et al. (2005). Exploration of the function and organization of the yeast early secretory pathway through an epistatic miniarray profile. Cell *123*, 507–519.

Schulz, T.A., and Creutz, C.E. (2004). The tricalbin C2 domains: lipid-binding properties of a novel, synaptotagmin-like yeast protein family. Biochemistry *43*, 3987–3995.

Sclip, A., Bacaj, T., Giam, L.R., and Südhof, T.C. (2016). Extended synaptotagmin (ESyt) triple knock-out mice are viable and fertile without obvious endoplasmic reticulum dysfunction. PLoS One *11*, e0158295.

Segawa, K., and Nagata, S. (2015). An apoptotic "eat me" signal: phosphatidylserine exposure. Trends Cell Biol. *25*, 639–650.

Sikorski, R.S., and Hieter, P. (1989). A system of shuttle vectors and yeast host strains designed for efficient manipulation of DNA in Saccharomyces cerevisiae. Genetics *122*, 19–27.

Suzuki, J., Umeda, M., Sims, P.J., and Nagata, S. (2010). Calcium-dependent phospholipid scrambling by TMEM16F. Nature *468*, 834–838.

Tong, A.H.Y., and Boone, C. (2007). 16 High-Throughput Strain Construction and Systematic Synthetic Lethal Screening in Saccharomyces cerevisiae. In Methods in Microbiology (Elsevier), pp. 369–386, 706–707.

Tong, A.H., Evangelista, M., Parsons, A.B., Xu, H., Bader, G.D., Pagé, N., Robinson, M., Raghibizadeh, S., Hogue, C.W., Bussey, H., Andrews, B., Tyers, M., and Boone, C. (2001). Systematic genetic analysis with ordered arrays of yeast deletion mutants. Science *294*, 2364–2368.

Toulmay, A., and Prinz, W.A. (2012). A conserved membrane-binding domain targets proteins to organelle contact sites. J. Cell Sci. *125*, 49–58.

Tremblay, M.G., and Moss, T. (2016). Loss of all 3 Extended synaptotagmins does not affect normal mouse development, viability or fertility. Cell Cycle *15*, 2360–2366.

Valm, A.M., Cohen, S., Legant, W.R., Melunis, J., Hershberg, U., Wait, E., Cohen, A.R., Davidson, M.W., Betzig, E., and Lippincott-Schwartz, J. (2017). Applying systems-level spectral imaging and analysis to reveal the organelle interactome. Nature *546*, 162–167.

Valverde, D.P., Yu, S., Boggavarapu, V., Kumar, N., Lees, J.A., Walz, T., Reinisch, K.M., and Melia, T.J. (2019). ATG2 transports lipids to promote autophagosome biogenesis. J. Cell Biol. *218*, 1787–1798.

Voeltz, G.K., Prinz, W.A., Shibata, Y., Rist, J.M., and Rapoport, T.A. (2006). A class of membrane proteins shaping the tubular endoplasmic reticulum. Cell *124*, 573–586.

Voss, C., Lahiri, S., Young, B.P., Loewen, C.J., and Prinz, W.A. (2012). ER-shaping proteins facilitate lipid exchange between the ER and mitochondria in S. cerevisiae. J. Cell Sci. *125*, 4791–4799.

Wach, A., Brachat, A., Pöhlmann, R., and Philippsen, P. (1994). New heterologous modules for classical or PCR-based gene disruptions in *Saccharomyces cerevisiae*. Yeast *10*, 1793–1808.

West, M., Zurek, N., Hoenger, A., and Voeltz, G.K. (2011). A 3D analysis of yeast ER structure reveals how ER domains are organized by membrane curvature. J. Cell Biol. *193*, 333–346.

Wolf, W., Kilic, A., Schrul, B., Lorenz, H., Schwappach, B., and Seedorf, M. (2012). Yeast Ist2 recruits the endoplasmic reticulum to the plasma membrane and creates a ribosome-free membrane microcompartment. PLoS One *7*, e39703.

Wong, L.H., Čopič, A., and Levine, T.P. (2017). Advances on the transfer of lipids by lipid transfer proteins. Trends Biochem. Sci. *42*, 516–530.

Yu, H., Liu, Y., Gulbranson, D.R., Paine, A., Rathore, S.S., and Shen, J. (2016). Extended synaptotagmins are Ca^{2+}-dependent lipid transfer proteins at membrane contact sites. Proc. Natl. Acad. Sci. USA *113*, 4362–4367.

STAR★METHODS

KEY RESOURCES TABLE

REAGENT or RESOURCE	SOURCE	IDENTIFIER
Antibodies		
Anti-GFP-HRP	Miltenyi Biotec	Cat# 130-091-833; RRID: AB_247003
Mouse Anti-Yeast PGK1	Thermo Fisher Scientific	Cat# 459250; RRID: AB_2532235
Goat Anti-Mouse HRP	Agilent Dako	Cat# P0447; RRID: AB_2617137
Bacterial and Virus Strains		
DH5α calcium-competent bacteria		N/A
Chemicals, Peptides, and Recombinant Proteins		
TetraSpeck microspheres 50 nm	Invitrogen / Thermo Fisher Scientific	Custom order
Lowicryl HM20 embedding kit	Polysciences, Inc.	Cat # 15924
Dextran from Leuconostoc spp. Mr ~40000	Sigma-Aldrich	Cat # 68084-25G
Acetone, anhydrous max 0.01 % water	VWR	Product code 83683.230
Hygromycin B solution	Scientific Laboratory Supplies, Corning	Cat # 30-240-CR
Nourseothricin solution	Jena Bioscience, LEXSY	Cat # AB-101L
Geneticin G418 Sulfate	Life Technologies Ltd	Cat # 11811-031
Ampicillin Sodium	Formedium Ltd	Cat # AMP10
S-(2-Aminoethyl)-L-cysteine hydrochloride	Sigma Aldrich	Cat # A2636-1G
L-Canavanine sulfate salt	Sigma Aldrich	Cat # C9758-1G
Concanavalin A	Sigma-Aldrich	Cat # C2010-25mg
Q5 High Fidelity DNA Polymerase	New England Biolabs	Cat # M0491S
Deposited Data		
Electron tomogram shown in Figure 2A	This study	EMDB: EMD-10287
Electron tomogram shown in Figure 2B	This study	EMDB: EMD-10299
Electron tomogram shown in Figure 2C	This study	EMDB: EMD-10300
Electron tomogram shown in Figure 2F	This study	EMDB: EMD-10301
Electron tomogram shown in Figure 2G	This study	EMDB: EMD-10302
Electron tomogram shown in Figure 2H	This study	EMDB: EMD-10303
Electron tomogram shown in Figure 3C	This study	EMDB: EMD-10304
Electron tomogram shown in Figure 3E	This study	EMDB: EMD-10306
Electron cryo-tomogram shown in Figure 5A	This study	EMDB: EMD-10308
Electron cryo-tomogram shown in Figure 5C	This study	EMDB: EMD-10309
Electron cryo-tomogram shown in Figure 6C and D	This study	EMDB: EMD-10310
Electron tomographic tilt series, related to EMD-10308	This study	EMPIAR: EMPIAR-10320
Electron tomographic tilt series, related to EMD-10309	This study	EMPIAR: EMPIAR-10321
Electron tomographic tilt series, related to EMD-10310	This study	EMPIAR: EMPIAR-10322
Experimental Models: Organisms/Strains		
S. cerevisiae strain: MATa, *his3-Δ200*, *leu2-3,112*, *ura3-52*, *lys2-801*, S288C background	Marko Kaksonen's lab	MKY0100
S. cerevisiae strain: MATα, *his3-Δ200*, *leu2-3,112*, *ura3-52*, *lys2-801*, S288C background	Marko Kaksonen's lab	MKY0102

(*Continued on next page*)

Continued

REAGENT or RESOURCE	SOURCE	IDENTIFIER
S. cerevisiae strain: MATa/MATα, *his3-Δ200/his3-Δ200*, *leu2-3,112::GAL1pr-I-SCEI-natNT2/leu2-3,112*, *ura3-52/ura3-52*, *lys2-801/lys2-801*	Marko Kaksonen's lab	MKY1604
S. cerevisiae strain: MATα, *his3-Δ200*, *leu2-3,112*, *ura3-52*, *lys2-801*, *TCB3-EGFP::HIS3MX6*	This study	WKY0117
S. cerevisiae strain: MATα, *his3-Δ200*, *ura3-52*, *lys2-801*, *sfGFP-SCS2*, not checked for *leu2-3,112::GAL1pr-I-SCEI-natNT2*	This study	WKY0133
S. cerevisiae strain: MATα, *his3-Δ200*, *ura3-52*, *lys2-801*, *sfGFP-IST2*, not checked for *leu2-3,112::GAL1pr-I-SCEI-natNT2*	This study	WKY0164
S. cerevisiae strain: MATα, *his3-Δ200, ura3-52, lys2-801, sfGFP-SCS2, TCB3-mRuby2::kanMX*, not checked for *leu2-3,112::GAL1pr-I-SCEI-natNT2*	This study	WKY0229
S. cerevisiae strain: MATα, *his3-Δ200, ura3-52, lys2-801, sfGFP-IST2, TCB3-mRuby2::kanMX*, not checked for *leu2-3,112::GAL1pr-I-SCEI-natNT2*	This study	WKY0230
S. cerevisiae strain: MATα, *his3-Δ200*, *leu2-3,112*, *ura3-52*, *lys2-801*, *TCB1-EGFP::HIS3MX6*, *TCB3-mRuby2::kanMX*	This study	WKY0231
S. cerevisiae strain: MATa, *his3-Δ200, leu2-3,112, ura3-52, lys2-801*, *SCS2Δ::URA3*, *SCS22Δ::URA3*, *IST2Δ::natNT2*	This study	WKY0254
S. cerevisiae strain: MATa, *his3-Δ200, leu2-3,112, ura3-52, lys2-801*, *SCS2Δ::URA3*, *SCS22Δ::URA3*, *IST2Δ::natNT2, TCB3-EGFP::HIS3MX6*	This study	WKY0255
S. cerevisiae strain: MATa, *his3-Δ200, leu2-3,112, ura3-52, lys2-801*, *TCB1Δ::URA3*, *TCB2Δ::hphNT1*, *TCB3Δ::hphNT1*	This study	WKY0280
S. cerevisiae strain: MATα, *his3-Δ200, leu2-3,112, lys2-801, ura3-52*, *SCS2Δ::URA3*, *SCS22Δ::URA3*, *TCB1Δ::hphNT1*, *TCB2Δ::hphNT1*, *TCB3Δ::hphNT1*, *IST2Δ::natNT2*	This study	WKY0297
S. cerevisiae strain: MATα, *his3-Δ200, ura3-52, lys2-801*, *SCS2Δ::URA3*, *SCS22Δ::URA3*, *TCB1Δ::kanMX6*, *TCB2Δ::hphNT1*, *TCB3Δ::hphNT1, sfGFP-IST2*, not checked for *leu2-3,112::GAL1pr-I-SCEI-natNT2*	This study	WKY0307
S. cerevisiae strain: MATα, *his3-Δ200, leu2-3,112, ura3-52, lys2-801*, *SCS2Δ::URA3*, *SCS22Δ::URA3*, *TCB1Δ::hphNT1*, *TCB2Δ::hphNT1*, *IST2Δ::kanMX6*, *kanMX6::GAL1pr-TCB3*-EGFP*::HIS3MX6*	This study	WKY0308
S. cerevisiae strain: MATα, *his3-Δ200, leu2-3,112, ura3-52, lys2-801*, *TCB3*-EGFP*::HIS3MX6*, *RTN1Δ::kanMX6*, *YOP1Δ::hphNT1*	This study	WKY0314
S. cerevisiae strain: MATα, *his3-Δ200, leu2-3,112, ura3-52, lys2-801*, *RTN1Δ::kanMX6*, *YOP1Δ::hphNT1*	This study	WKY0320
S. cerevisiae strain: MATα, *his3-Δ200, ura3-52, lys2-801*, *RTN1Δ::kanMX6*, *YOP1Δ::hphNT1, sfGFP-SCS2*, not checked for *leu2-3,112::GAL1pr-I-SCEI-natNT2*	This study	WKY0322
S. cerevisiae strain: MATα, *his3-Δ200, ura3-52, lys2-801*, *RTN1Δ::kanMX6*, *YOP1Δ::hphNT1, sfGFP-IST2*, not checked for *leu2-3,112::GAL1pr-I-SCEI-natNT2*	This study	WKY0323

(*Continued on next page*)

Continued

REAGENT or RESOURCE	SOURCE	IDENTIFIER
S. cerevisiae strain: MATa, *can1Δ::STE2pr-his5*, *lyp1Δ*, *ura3Δ0*, *leu2Δ0*, *his3Δ1*, *met15Δ0*	Tong and Boone, 2007	Y7092
S. cerevisiae strain: MATα, *can1Δ::STE2pr-his5*, *lyp1Δ*, *ura3Δ0*, *leu2Δ0*, *his3Δ1*, *met15Δ0*, *TCB1Δ::natNT2*, *TCB2Δ::hphNT1*	This study	WKY0330
S. cerevisiae strain: MATα, *can1Δ::STE2pr-his5*, *lyp1Δ*, *ura3Δ0*, *leu2Δ0*, *his3Δ1*, *met15Δ0*, *TCB1Δ::natNT2*, *TCB2Δ::hphNT1*, *TCB3Δ::LEU2*	This study	WKY0331
S. cerevisiae strain: MATα, *his3-Δ200, leu2-3,112, ura3-52, lys2-801*, *RTN1Δ::kanMX6*, *YOP1Δ::hphNT1*, *TCB2-EGFP::HIS3MX6*	This study	WKY0335
S. cerevisiae strain: MATa, *his3-Δ200, leu2-3,112, lys2-801*, *ura3Δ::GPDpr-GCaMP6f-ADHt-kanMX4*, *SCS2Δ::URA3*, *SCS22Δ::URA3*, *IST2Δ::natNT2*	This study	WKY0366
S. cerevisiae strain: MATa, *his3-Δ200, leu2-3,112, ura3-52, lys2-801*, *TCB1Δ::URA3*, *TCB2Δ::hphNT1*, *TCB3Δ::hphNT1*, *NVJ1Δ:: kanMX6*	This study	WKY0412
Yeast Deletion Clones, MAT-A Complete set	Thermo Fisher	Cat. no. 95401.H2
Yeast DAmP Library MATa	Gift from Maya Schuldiner<br>Schuldiner et al., 2005	N/A
**Oligonucleotides**		
Primers, see Table S2	This study	N/A
**Recombinant DNA**		
Plasmid: pFA6a-EGFP-HIS3MX6	Janke et al., 2004	pYM28
Plasmid: pYM-N-sfGFPΔC-I-SceI^{site}-CYC1 term-URA3-TEF1pr-I-SceI^{site}-sfGFP	Khmelinskii et al., 2011	pMaM173
Plasmid: pFA6a-natNT2	Janke et al., 2004	N/A
Plasmid: pFA6a-hphNT1	Janke et al., 2004	N/A
Plasmid: pFA6a-klURA3	Delneri et al., 1999	N/A
Plasmid: pFA6a-Leu2	Longtine et al., 1998	N/A
Plasmid: pFA6a-KanMX6	Wach et al., 1994	N/A
Plasmid: pFA6a-KanMX6-GAL1pr	Longtine et al., 1998	N/A
Plasmid: pRS425-2μ-LEU2-SEC63-mRFP	Addgene (Metzger et al., 2008)	pSM1959<br>http://n2t.net/addgene:41837
Plasmid: pFA6a-link-yomRuby2-KanMX	Addgene (Lee et al., 2013)	http://n2t.net/addgene:44953
Plasmid: pFA6a-yomRuby2-KanMX	This study	pWK0027
Plasmid: pRS316-URA3	Sikorski, and Hieter, 1989	pRS316
Plasmid: pRS316-Tcb3-EGFP	This study	pWK092
Plasmid: pRS316-chimeraN-Tcb3-EGFP	This study	pWK119
Plasmid: pRS316-Tcb3-ΔSMP-EGFP	This study	pWK096
Plasmid: pRS316-chimeraC-Tcb3-EGFP	This study	pWK120
Plasmid: pRS316-chimeraN-Tcb3-ΔSMP-EGFP	This study	pWK140
Plasmid: pWJ1512	Reid et al., 2011	pWJ1512
Plasmid: pRS415-prCUP1-Tcb3-ΔSMP-EGFP	This study	pWK142
Plasmid: pRS306K-GPD1p-GCaMP6f-ADH1t-a	Carbó et al., 2017	PSAB367
gBlocks: $(GGGGS)_5$-2x-Ist2$_{PB}$	Integrated DNA Technologies	N/A
**Software and Algorithms**		
RELION	Bharat et al., 2015<br>Scheres, 2012	http://www2.mrc-lmb.cam.ac.uk/relion
MATLAB-based correlation scripts	Kukulski et al., 2011<br>Schorb et al., 2017	https://www.embl.de/download/briggs/cryoCLEM/index.htm
MATLAB-based distance measurement scripts	This study	https://gitlab.com/jboulanger/psurfdist

(*Continued on next page*)

Continued		
REAGENT or RESOURCE	SOURCE	IDENTIFIER
SerialEM	Mastronarde, 2005	http://bio3d.colorado.edu/SerialEM/
IMOD	Kremer et al., 1996	http://bio3d.colorado.edu/imod/
GraphPad Prism	Graphpad	https://www.graphpad.com
Fiji	Schindelin et al., 2012	https://imagej.net/Fiji
Icy	de Chaumont et al., 2012	http://icy.bioimageanalysis.org
eC-CLEM	Paul-Gilloteaux et al., 2017	http://icy.bioimageanalysis.org/plugin/ec-CLEM
Other		
Quantifoil EM grids (copper, 200 mesh, R2/2)	www.quantifoil.com	No product code
UltrAuFoil EM grids (gold, 200 mesh, R2/2)	www.quantifoil.com	No product code
Aluminium specimen carrier type A	Wohlwend GmbH, Sennwald, Switzerland	Art. # 241
Aluminium specimen carrier type B	Wohlwend GmbH, Sennwald, Switzerland	Art. # 242
Carbon film EM grids (copper, 200 mesh)	Agar Scientific	Code AGS160

LEAD CONTACT AND MATERIALS AVAILABILITY

Further information and requests for resources and reagents should be directed to and will be fulfilled by the Lead Contact, Wanda Kukulski (kukulski@mrc-lmb.cam.ac.uk). Plasmids and yeast strains will be available upon request with a completed Materials Transfer Agreement.

EXPERIMENTAL MODEL AND SUBJECT DETAILS

Yeast Strains

Chemically-competent yeast cells were prepared from cultures grown in standard YPD medium at 30°C. Yeast cultures for fluorescence live imaging or EM sample preparation were inoculated from overnight pre-cultures and grown to mid-log phase at 25°C in 5 - 50 ml strain-specific selective dropout medium without tryptophan. All strains were grown with 2% glucose as carbon source with exception of WKY0308, the galactose-inducible Tcb3-EGFP 5Δ strain, which was grown in synthetic complete media lacking tryptophan (SC-Trp) with 1% raffinose and induced by addition of 1% galactose.

METHOD DETAILS

Yeast Genetic Techniques

Yeast strains (see Key Resources Table) were generated by homologous recombination of the target genes with PCR cassettes for gene deletion or tagging as described in (Janke et al., 2004). Seamless N-terminal tagging with sfGFP was performed using the pMaM173 plasmid and yeast strains with *GAL1-I-SCEI* integration to loop out the URA marker as described in (Khmelinskii et al., 2011). Individual transformants were isolated on selection plates and insertion of PCR cassettes was verified by colony PCR. Yeast strains carrying multiple deletions were generated either by subsequent homologous recombination or by mating and tetrad dissection. The galactose-inducible Tcb3-EGFP strain was generated by insertion of the KanMX6-GAL1pr PCR cassette amplified from pFA6a-KanMX6-GAL1pr, which introduced the *GAL1* promoter 5' of the endogenous *TCB3* locus. By mating and tetrad dissection, a galactose-inducible Tcb3-EGFP strain deleted for *tcb1/2 scs2/22 ist2* was isolated. The GCaMP reporter strain deleted for *scs2/22 ist2* was generated by homologous recombination of the PvuII linearized PSAB367 plasmid (Carbó et al., 2017) at the *URA3* locus and selected for on G418 selection plates. All yeast transformations were performed according to standard lithium acetate transformation protocols.

Plasmid Constructs and Cloning

The *TCB3* gene including 500 bp of the 5' UTR and 100 bp of the 3' UTR was amplified from purified genomic DNA using Phusion DNA polymerase (New England Biolabs). 5' Sal1 and 3' Sac1 restriction sites were introduced with amplification primers. Amplified DNA was ligated with linearized pRS316 plasmid at Sal1 and Sac1 restriction sites. A C-terminal EGFP cassette was introduced by homologous recombination in yeast of the amplified EGFP sequence with homology arms for the 3' end of *TCB3* and 3' untranslated region of the Msc1 linearized pRS316-Tcb3-URA3 vector. ChimeraN-Tcb3-GFP was generated by homologous recombination of Afe1 linearized pRS316-Tcb3-GFP and PCR-amplified sequence of the N-terminal part of *ERG11* including a transmembrane domain (1-55 aa-3x-gly) with homology arms directly targeting the regions before the start codon and 742 bp into the sequence of *TCB3*. This resulted in replacement of the N-terminal 247 aa of Tcb3. The SMP domain (272-479 aa) of Tcb3 was replaced by homologous

recombination with a synthetic DNA primer containing a $(GGGGS)_2$ linker sequence and homology arms targeting between 813 bp and 1438 bp into the *TCB3* sequence of ClaI linearized pRS316-Tcb3-GFP. To generate the chimeraC-Tcb3-GFP plasmid, a synthetic sequence containing a $(GGGGS)_5$ linker and 2x the polybasic PM-binding region of Ist2 (929-946 aa) (gBlocks, Integrated DNA Technologies) with homology arms was integrated 1467 bp into the sequence of *TCB3* and before the EGFP sequence into Age1/BamH1 linearized pRS316-Tcb3-EGFP. ChimeraN-ΔSMP-Tcb3-GFP was generated by replacing the SMP domain in pRS316-chimeraN-Tcb3-GFP with a synthetic linker as described above. The copper-titratable pRS415-prCUP1-Tcb3-ΔSMP-GFP construct was generated directly in the *tcb1/2/3Δ nvj1Δ* yeast strain by homologous recombination of Hpa1 linearized pWJ1512 with PCR-amplified Tcb3-ΔSMP-GFP with adaptamer A and B overlaps introduced through the amplification primers (Reid et al., 2011).

For C-terminal tagging with mRuby2, we removed the 5' linker of the mRuby2 sequence from pFA6a-link-yomRuby2-KanMX (Lee et al., 2013). A Sal1 restriction site was introduced directly 5' of the mRuby2 sequence and the S3 primer binding site. Following selection, all plasmids were recovered from construct-bearing yeast strains by cell lysis with glass-beads and plasmid prep with the QIAprep Spin Miniprep Kit. Plasmids were re-transformed into calcium-competent bacteria for amplification. Correct insertion and sequence for all inserts except pRS415-prCUP1-Tcb3-ΔSMP-GFP was validated by DNA sequencing (GATC Biotech).

Live-cell Imaging

Yeast cells were grown to mid-log phase in SC-Trp or strain-specific selective dropout medium lacking tryptophan at 25°C. Cells were adhered to glass coverslips using Concanavalin A and imaged in SC-Trp medium using a Nikon TE2000-E widefield microscope, which was controlled by Nikon NIS Elements 4.4 and equipped with 100x oil-immersion TIRF objective with NA 1.49, NEO sCMOS DC-152Q-C00-FI camera (Andor), Lambda DG-4 lamp (Sutter Instruments), filter sets 49002 ET GFP (Chroma), excitation 470/40, dichroic T495lpxr, emission 525/50 for GFP, 49005 ET DSRED (Chroma), excitation 545/30, dichroic T570lp, emission 620/60 with additional emission filter 605/70 for RFP, 49006 ET CY5 (Chroma), excitation 620/60, dichroic T660lpxr, emission 700/75 for far red. Images were acquired with 0.5-3 s exposure time, depending on fluorescence intensity of target proteins. For some experiments, cells were imaged using a Nikon Ti2 widefield microscope setup similar to the one described above, except that a Niji LED light source (bluebox optics) was used. For GFP and RFP excitation, filters ET470/40x and ET545/30x, respectively, in the Niji box were used. Representative images used in Figure panels were adjusted individually for best contrast and brightness in Fiji and therefore do not represent quantitative differences in fluorescence intensity, with exception of Figures S2B, S2D, and S3C, which were adjusted for quantitative comparison. Line profiles to visualize colocalization were extracted around the cell cortex and straightened using Fiji.

SGA Analysis

Synthetic Genetic Array (SGA) analysis was performed according to (Tong and Boone, 2007) using a colony pinning robot (Singer Instruments). The *tcb1/2Δ* query strain (WKY0330) or *tcb1/2/3Δ* query strain (WKY0331) without or with Tcb3 rescue constructs were mated to the haploid deletion mutant array and Decreased Abundance by mRNA Perturbation (DAmP) array at a density of 384 spots per plate. Diploid strains were selected, then pinned onto sporulation medium and incubated for 5-7 days at 25°C. Haploid *MATa*, triple mutants (for the *tcb1/2Δ* query strain), quadruple mutants (for the *tcb1/2/3Δ* query strain), and rescue mutant strains were selected on appropriate selection media. Plate supplements were used at the following concentrations: 50 mg/L Canavanine, 50 mg/L S-Aminoethyl-L-cysteine, 200 mg/L G418, 100 mg/L ClonNAT, 300 mg/L HygromycinB. Synthetic sick and lethal phenotypes were identified using PhenoBooth plate imager (Singer Instruments) and PhenoSuite colony counting and analysis software (Singer Instruments). Colony growth on selection plates was scored and compared to colony growth on *MATa* control plates. Colony size was normalized per plate, row and column and a $\log_2$ ratio was calculated. Reduction of growth by factor of 2 ($\log_2$ ratio less than -1.00) was used as a cut-off for synthetic sick phenotypes. To verify selected hits, quadruple mutants in the *tcb1/2/3Δ* background were isolated by tetrad dissection or generated anew by homologous recombination with gene deletion cassettes and subsequent verification. For serial dilution growth assays, late log-phase cultures were adjusted to OD_{600} = 1.0 before preparing 10-fold serial dilutions. A volume of 3 μl per dilution was spotted on plates. Because Rim101 pathway mutants are sensitive to growth under high salt conditions (Hayashi et al., 2005), growth of these mutants was assayed on YPD with 0.1 M LiCl. Serial dilution growth assays of *tcb1/2/3Δ nvj1Δ* cells transformed with the pRS415-prCUP1-Tcb3-ΔSMP-GFP rescue plasmid were performed on YPD plates with 0.5 μM, 5 μM and 20 μM $CuSO_2$. Cells grown on YPD plates without $CuSO_2$ showed accumulation of fluorescent ER signal in a subset of cells after 2 days of growth on plate, likely due to leaky expression of Tcb3-ΔSMP-GFP from the *CUP1* promoter under these conditions.

Western Blot

About 20 OD_{600} units of yeast cells were pelleted by centrifugation and lysed for 10 min at 4°C with glass beads in 10 μl of lysis buffer per OD_{600} unit (25 mM Tris pH 7.4, 150 mM NaCl, 1% Triton X-100, 0.1% SDS, protease inhibitor cocktail from New England Biolabs). The lysates were cleared for 10 min at 13900x g at 4°C and the protein concentrations were determined by BCA Protein assay (Thermo Fisher). A total of 30 μg, or 6 μg, protein per lane and lysate in NuPAGE LDS sample buffer (Invitrogen) were run on NuPAGE 4-12% Bis-Tris gels (Invitrogen). Gels were then blotted onto PVDF membrane (GE Healthcare) by wet transfer. Membranes were blocked with 5% dried skimmed milk powder. Membranes were incubated with primary antibodies against GFP (Miltenyi Biotec, Cat# 130-091-833, HRP-coupled) or phosphoglycerate kinase (Thermo Fisher Scientific, Cat# 459250) and, if required, with a

secondary HRP-coupled antibody (Agilent Dako, Cat# P0447). Membranes were washed with PBS-T after each antibody incubation. The blots were developed using ECL Prime reagent (GE Healthcare) and Fuji Super RX X-ray films.

Room-temperature Correlative Light and Electron Microscopy

Room-temperature (RT-)CLEM was performed as described in (Kukulski et al., 2011) with modifications described in (Ader and Kukulski, 2017). Yeast cells were grown to mid-log phase in SC-Trp medium or strain-specific selective dropout medium as described above and pelleted by vacuum filtration. Resulting yeast paste was high-pressure frozen in 200 μm deep wells of aluminium carriers (Wohlwend) using a HPM100 (Leica Microsystems). Freeze-substitution and Lowicryl HM20 (Polysciences, Inc.) resin-embedding followed the protocol and timing described in (Kukulski et al., 2011), except that 0.03-0.05% uranyl acetate in acetone was used for freeze-substitution. During some preparations, samples were shaken on dry ice for the first 2 h of freeze-substitution. Sections of 270 nm thickness were cut with an Ultra 45° diamond knife (Diatome) on an Ultracut E microtome (Reichert). The sections were floated onto PBS and picked up with 200 mesh carbon-coated copper grids (AGS160, Agar Scientific). Fluorescent TetraSpeck beads (Invitrogen), 50 nm in diameter, were adsorbed onto the grid. Directly after sectioning, grids were mounted for fluorescence microscopy in PBS and imaged as described above for live-cell imaging. Prior to ET, 15 nm gold beads (Electron Microscopy Sciences) were adsorbed on the sections, which were then post-stained for 10 min with lead citrate. Scanning transmission EM tomography was done on a TF20 microscope (FEI) with an axial brightfield detector, using a camera length of 200 mm and a 50 μm C2 aperture (Ader and Kukulski, 2017; Hohmann-Marriott et al., 2009). For low magnification correlation either montages or tilt series at 4.4 nm or 3.4 nm pixel size were acquired using SerialEM (+/- 60° tilt range, 2° increment, single axis acquisition) (Mastronarde, 2005). Higher magnification dual axis tilt series were acquired with 1° increment and at 1.1 nm pixel size (Mastronarde, 1997). All tomographic reconstructions were done in IMOD (Kremer et al., 1996) and fiducial-based correlation was done using MATLAB-based scripts described in (Kukulski et al., 2011).

Classification and Quantitative analysis of ER-PM Contacts Identified by RT-CLEM

In tomograms of resin-embedded cells, ER morphologies at ER-PM contacts associated with signals of the different GFP-tagged proteins were visually classified into four different classes. The first class contained fluorescent signals spread over ER sheets or cisternae ("flat ER sheet"). In the second class, fluorescent signals localized to ER tubes ("tubular ER"). Isolated punctate fluorescent signals localizing to curved ends of ER sheets or fenestrations were also included in this class. ER of more complex morphology, which contained both tubular and flat parts throughout the volume, were counted as a third class ("mixed flat & tubular"). Fluorescent signals that could not be assigned to any ER membrane were classified as "not assigned" (n.a.). "Number of observations" refers to the number of individual, continuous fluorescent signals, correlated to individual ER cisternae, assigned to each class. To show the 3D membrane morphology in the figure panels, segmentations of the cER membrane, along the cytosolic leaflet, throughout the tomographic volume were done in Amira (Thermo Fisher). The segmentation models in figures are displayed tilted and rotated for better visualization of the cER morphology. In total, the RT-CLEM dataset on sfGFP-Scs2 cells consisted of 35 correlated fluorescent signals in 15 cells, on sfGFP-Ist2 cells 42 correlated signals in 14 cells, on Tcb3-EGFP cells 46 correlated signals in 18 cells, on Tcb3-EGFP *scs2/22*Δ *ist2*Δ cells 46 correlated signals in 21 cells, on sfGFP-Ist2 *tcb1/2/3*Δ *scs2/22*Δ cells 63 correlated signals in 25 cells, on Tcb3-EGFP *rtn1*Δ *yop1*Δ cells 33 correlated signals in 12 cells and on plasmid-expressed Sec63-RFP *tcb1/2/3*Δ *scs2/22*Δ *ist2*Δ cells 17 correlated signals in 13 cells. Resin sections from different freeze-substitution and embedding runs varied in preservation quality, which resulted in variances in fluorescence and visibility of membrane ultrastructure. Therefore, correlations to areas with poor preservation of membrane ultrastructure were excluded from intermembrane distance measurements, which is why the number of sites and cells analyzed for intermembrane distance measurements differ from those for morphology classification. Intermembrane distances at ER-PM contacts were measured approx. 100 nm around the extent of fluorescent signals, according to the transformation based on fiducial markers. Points along the cytosolic leaflets of the ER membrane and of the PM were clicked in IMOD where the membrane was best visible, approx. every 10 nm in the x/y plane. The membranes were traced every 5.5 nm in z direction throughout the tomographic volume. Using MATLAB, orientation of the point clouds were determined by fitting planes and rotating them so that bi-harmonic spline interpolations enabled to recover the underlying smooth surface as a regular grid of 20 x 20 points. The minimum and maximum distance, the average distance and standard deviation were calculated between the PM surface model and the sets of points clicked at the ER membrane. The output of the surface model was plotted and visually inspected using MATLAB. In general, one individual distance measurement (N), corresponded to one ER-PM contact associated with one individual, continuous fluorescent signal. In some instances, the fluorescent signal was associated with a complex cER morphology, which required further division into 2 or 3 sites of measurement, to prevent distortions of the surface modeling by extreme changes in membrane morphology. These sites were counted as individual distance measurements. The average and the standard deviation (SD) of all individual measurements for each analyzed yeast strain were combined and compared.

Cryo-EM Sample Preparation

Yeast cells were grown to mid-log phase in SC-Trp medium. One OD_{600} unit of cells was pelleted by centrifugation at 3000x g. Cells were resuspended in 500 μl SC-Trp containing 15% high-molecular weight dextran (w/v) and 2% glucose, or 1% raffinose for cells with induced protein expression. Vitrification by plunge-freezing was done with a manual plunger and a temperature controlled liquid ethane reservoir at -181°C (Russo et al., 2016). 5 μl cell suspension were applied to Quantifoil R2/2 Cu 200 mesh or UltrAuFoil R2/2 Au

200 mesh grids, which beforehand were plasma treated for 40 s using Fischione 1070 Nano Clean with 9/1 argon/oxygen mix and 38 W power. Grids were backside-blotted for 12-15 s with filter paper (Whatman No. 1) immediately before plunge-freezing.

Cryo-FIB Milling

Cryo-FIB milling of plunge-frozen yeast cells was performed on a Scios DualBeam FIB/SEM microscope (FEI) equipped with a Quorum PP3010T cryo-FIB/SEM preparation system. The loading stage and milling procedure were adapted, with minor alterations, from (Schaffer et al., 2015). The temperature of the cryo-stage was kept between -170°C to -180°C and the anti-contaminator temperatures below -190°C. Grids were sputter-coated with platinum in the Quorum preparation system for 60 s at 10 mA current before milling. Additionally, grids were coated with a layer of organometallic platinum using the gas injection system for 30 s at a distance of 5 mm with a stage tilt of 25°. Imaging settings for the electron beam were 2 kV voltage and 13 pA current and for the ion beam 30 kV voltage and 10 pA current. Initial rough milling was performed with 30 kV ion beam voltage and 0.5 or 1 nA current at a stage tilt of 35°-40°, below and above the cells of interest. The stage was then tilted to 17° and cells were subsequently milled to 3 μm lamella thickness with 30 kV, 0.3 nA, then to 1 μm lamella thickness with 0.1 nA. Usually 2-3 lamellae were rough-milled per grid before proceeding to a fine milling step. Fine milling was performed by milling either at 16° or 18° stage tilt as described by (Schaffer et al., 2017) until the lamellae were approx. 300 nm thick. Final polishing of the lamellae was performed at 17° stage tilt and resulted in 150-300 nm thick lamellae with 10° pre-tilt relative to the grid. All fine milling steps were done at 16 kV voltage and 11 pA or 23 pA ion beam current to minimize potential ion beam damage to the lamellae.

Cryo-fluorescence Microscopy

To target yeast cells with high levels of intracellular Ca^{2+}, WKY0366 cells were resuspended in 500 μl SC-Trp containing 15% high molecular weight dextran (w/v), 2% glucose and 200 mM calcium chloride before plunge-freezing. Cells were plunge-frozen as described above, within a 2-10 min time window after resuspending in the calcium chloride containing medium. Cryo-fluorescence imaging of yeast cells on TEM grids was performed with few adaptations according to (Ader et al., 2019) on the Leica EM cryo-CLEM system with an HCX PL APO 50x cryo-objective with NA = 0.9 (Leica Microsystems), an Orca Flash 4.0 V2 SCMOS camera (Hamamatsu Photonics), a Sola Light Engine (Lumencor) and the L5 filter, excitation 480/40, dichroic 505, emission 527/30 for detection of GFP fluorescence. The room humidity was kept between 20-25% and the microscope stage was cooled to -195°C during imaging. Using the Leica LAS X software, a 1.5 x 1.5 mm tile scan z-stack with 18 μm range and 2 μm step size was recorded around the center of the grid in the GFP channel (17% intensity, 3 s exposure) and in the brightfield channel (30 intensity, 50 ms exposure). Autofocus routine was performed in the brightfield channel. Groups of cells with the highest fluorescent signals were identified by adjusting the contrast and brightness in Fiji. These groups of brightest cells were targeted for cryo-FIB milling by visual correlation using overview SEM images for identification of cells. Overlays of cryo-FM and cryo-EM images were generated using the eC-CLEM plugin in Icy (Paul-Gilloteaux et al., 2017).

Electron Cryo-tomography

Cryo-ET data acquisition was done on two Titan Krios microscopes (Thermo Fisher) fitted with Quantum energy filter and K2 direct electron detector (Gatan) used in counting mode, using SerialEM (Mastronarde, 2005). Montaged images of the central part of the grid were acquired at a pixel size of 200 nm to localize the lamellae on the grid. Overview montages of the individual lamellae were acquired at pixel sizes of 5.4 or 5.5 nm, depending on the microscope, to assess the lamellae quality and identify ER-PM contact sites within the cells. Tilt series were collected between ±60° starting from 0° using a grouped-dose symmetric tilt scheme with 1° increment and group size of 4 (Bharat et al., 2018; Hagen et al., 2017). Cryo-ET datasets were acquired during multiple sessions, during which the calibrated pixel size at which tilt series were acquired varied between 3.54 Å and 3.77 Å. For analysis of all datasets, the average pixel size 3.7 Å was used. The target dose rate was kept at ~2-4 e^{-}/pixel/s on the detector depending on lamella thickness. The energy filter slit width was set to 20 eV. The nominal defocus was varied between -3.5 μm and -6 μm for different tilt series. A dose of approximately 1.0-1.3 $e^{-}/Å^{2}$ was applied per image of tilt series. Tilt images were collected as frames, and frames were aligned using the alignframes program in IMOD. Tilt series were aligned using patch tracking and reconstructed using IMOD. The contrast transfer function was estimated and compensated for by phase flipping in IMOD. Tomograms were reconstructed in IMOD by backprojection for image processing (see below), and by simultaneous iterative reconstruction technique (SIRT) with 10 iterations at a pixel size of 7.4 Å for display in Figures. In addition, to improve visibility of low-resolution morphological features, nonlinear anisotropic diffusion filtering (Frangakis and Hegerl, 2001) in conjunction with gaussian filtering was applied to tomographic slices.

Image Processing and Classification of Cryo-ET Data

To analyze the density layer observed at the PM of ER-PM contacts at high cytosolic calcium, a subtomogram averaging approach described previously (Bharat et al., 2018) was adapted. The aim was to obtain 2D averages from subvolumes within each individual layer. First, the 4 (out of 10) tomograms of the high calcium dataset that displayed a visible density layer were rotated around their x axis in IMOD to display the PM perpendicularly to the viewing plane. Next, along the inner leaflet of the PM where a dense coat was visible, points were clicked in slices spaced by 8 nm in z direction. For comparison, the same was done at the PM outside the ER-PM contact site in the same tomograms. A spline was fitted through each set of points in MATLAB and subtomograms were extracted along the fitted spline in overlapping boxes using Relion (Bharat and Scheres, 2016). The in-plane rotation angle from the spline fit

was written out and retained to restrain subsequent alignment. The subtomograms were projected into 2D images and subjected to 2D averaging using Relion (Scheres, 2012) separately for each area of PM. To improve accuracy of our 2D averaging, we used the membrane profile of the cell to limit the in-plane angular rotation search in the alignment procedure. First, we empirically optimized the box sizes for alignment, since too large a box would lead to smearing of features and too small a box would have less signal from the protein and membranes, leading to potentially spurious alignments. Once we had selected an appropriate box size (444 Å), several 2D classifications were performed with different low pass filters and by tuning the T value in Relion. Selection of well-aligned 2D classes was based on careful visual examination of results from different runs, using features such as the lipid bilayer as a readout for good alignments. Final class averages from PM-ER contacts with the extra layer were compared to free PM for each tomogram individually, to minimize defocus effects on the appearance of the membrane bilayer that vary between tomograms. To measure the extent of the extra layer in the 4 tomograms of cells with high cytosolic calcium and in 5 tomograms of the control cells, the volume of cER and the volume occupied by the extra layer in the tomograms were segmented and calculated in Amira. The ratio of the extra layer volume to the volume of cER was determined and served as a measure of the extent of the extra layer.

To analyze the bridging particle densities, we aimed at classifying and averaging densities from multiple contact sites and several tomograms. Individual particle densities, which appeared to bridge the ER and PM in tomograms of *scs2/22Δ ist2Δ tcb1/2Δ* cells overexpressing Tcb3-GFP, were manually picked in IMOD by clicking one point at the ER membrane base, a second point at the PM, and two auxiliary points approx. 10 nm away from the bridging density along the ER membrane and along the PM. The set of points allowed calculating the in-plane angle relative to the membranes for each bridging density. These angles were used to calculate the difference from perpendicular orientation to the PM for each individual particle, as well as for subtomogram averaging. Subtomograms were extracted at the center of each density using Relion and the in-plane rotation angle for each subtomogram was written out and retained for constraining angular searches in alignment. Subtomograms were projected into 2D images and subjected to 2D classification and averaging in Relion. To determine robust parameters that would give consistent averaging results, we employed the following strategy: Several classifications with collapsed 2D images with varying numbers of classes were performed. We also varied the size of the circular mask and low pass filters. Furthermore, initially, we produced separate 2D averages from different tomograms separately. Visual assessment of results from different runs with a range of parameter sets revealed well-aligned classes, which were consistently similar in terms of the density arrangement, and thus resulted in the same structural interpretation. This gave us confidence to combine data and include particles from many contact sites and tomograms into a merged data set, resulting in the final 2D averages, which were qualitatively similar to ones obtained from individual tomograms. One representative run with 5 class averages, which encompasses the major heterogeneity detected in our data set, is shown in Figure 6. The dataset for 2D averaging of bridging densities in *scs2/22Δ ist2Δ tcb1/2Δ* cells overexpressing Tcb3-GFP consisted of 17 electron cryo-tomograms, from which 1513 bridging densities were selected based on their visibility approx. parallel to the viewing plane. Line profiles of class averages were made in Fiji along the major axes of the average particles over a width of 15 pixels, which included the whole width of the average particles.

Average ER-PM intermembrane distances in electron cryo-tomograms were measured similarly as described for the RT-CLEM dataset. However, each data point (N) corresponds to the average distance within a contact area extending over approximately 100-200 nm in x,y. We measured 21 contact areas in 10 Ca^{2+} treated cells and 22 contact areas in 10 untreated cells. For positions where ER membrane buckled towards PM, single local distances were measured manually at the shortest distance between the PM and the ER membrane using IMOD.

QUANTIFICATION AND STATISTICAL ANALYSIS

Quantitative analysis of membrane morphology and membrane distances in electron tomograms are described in the respective Methods sections which contain information about dataset size, mean, SD and N. Statistical significance was tested using Welch's unequal variances t-test in Graphpad Prism.

DATA AND CODE AVAILABILITY

Extended results of SGA analysis are available in Table S1. Full lists of yeast strains, primers and plasmids are available in the Key Resources Table and Table S2. Representative ET reconstructions corresponding to data shown in Figures 2A–2C, 2F–2H, 3C, 3D, 5A, 5C, 6C, and 6D, have been deposited in the Electron Microscopy Data Bank (EMDB) under the accession codes EMD-10287, EMD-10299, EMD-10300, EMD-10301, EMD-10302, EMD-10303, EMD-10304, EMD-10306, EMD-10308, EMD-10309 and EMD-10310. The tilt series corresponding to the cryo-ET reconstructions in EMD-10308, EMD-10309 and EMD-10310 have been deposited at the Electron Microscopy Public Image Archive (EMPIAR) under the accession codes EMPIAR-10320, EMPIAR-10321 and EMPIAR-10322, respectively.

Developmental Cell, Volume *51*

Supplemental Information

Tricalbins Contribute to Cellular Lipid Flux and Form Curved ER-PM Contacts that Are Bridged by Rod-Shaped Structures

Patrick C. Hoffmann, Tanmay A.M. Bharat, Michael R. Wozny, Jerome Boulanger, Elizabeth A. Miller, and Wanda Kukulski

Supplemental Figure S1. Related to Figure 3.

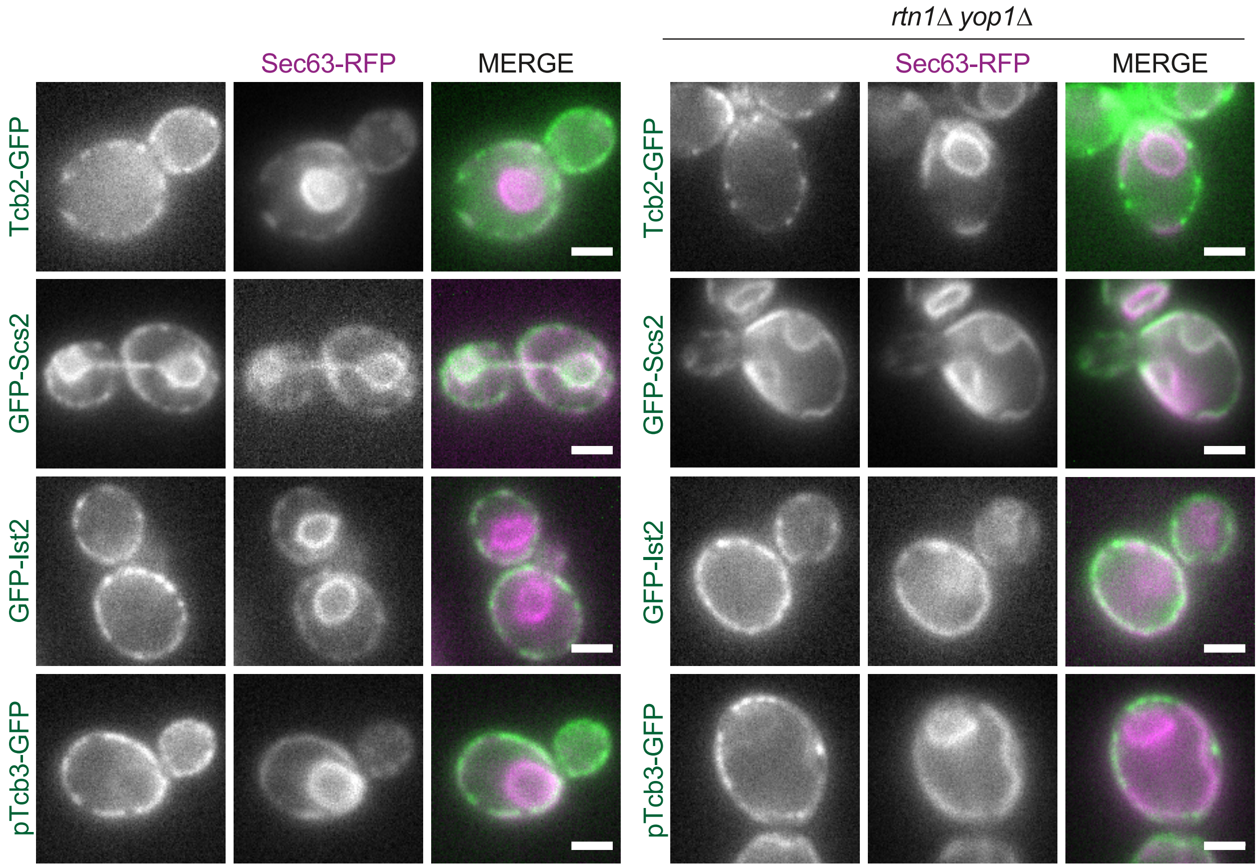

Supplemental Figure S1. Related to Figure 3. Reticulon-dependent localization of ER-PM bridging proteins

Live FM of wild type (left panel) and *rtn1*Δ *yop1*Δ (right panel) cells expressing either Tcb2-EFGP, GFP-Scs2, GFP-Ist2 or plasmid-encoded Tcb3-GFP, in combination with plasmid-encoded Sec63-RFP. Scale bars: 2 *μ*m.

Supplemental Figure S2. Related to Figure 4.

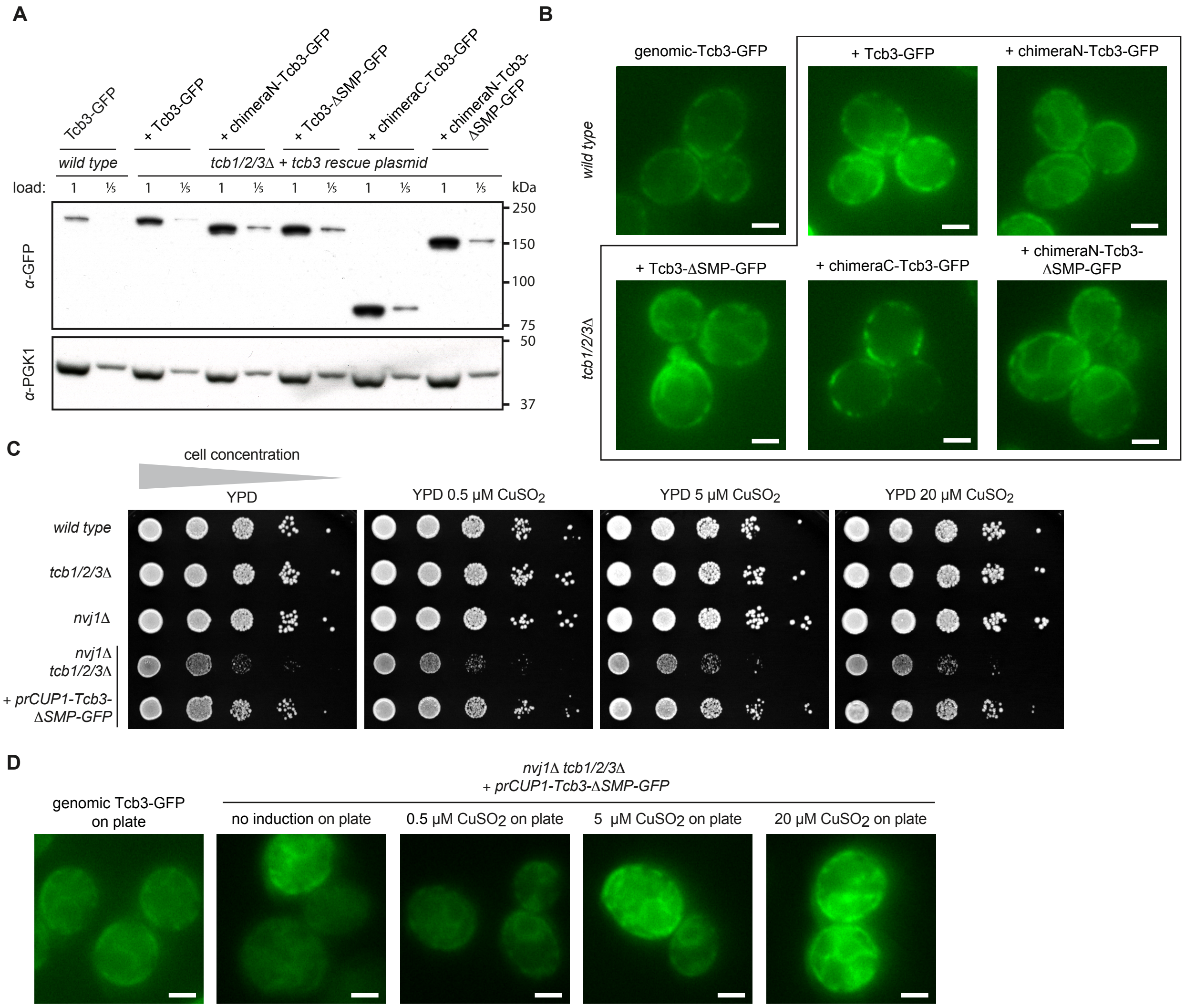

Supplemental Figure S2. Related to Figure 4. Protein expression levels of the rescue constructs used in the SGA

A: Western blot using an antibody against GFP, to compare levels of GFP-tagged Tcb3 at the genomic locus in wild type cells, with the different GFP-tagged Tcb3 rescue plasmids (see Figure 4) in the *tcb1/2/3*Δ query strain. For each sample 30 µg total protein (lanes labeled 1) and a five-fold dilution (lanes labeled 1/5) was loaded. Lower panel: Pgk1 was blotted for as loading control. **B:** Live FM of GFP-tagged Tcb3 at the genomic locus in wild type cells, and of the different GFP-tagged Tcb3 rescue plasmids in the *tcb1/2/3*Δ query strain, using the same imaging conditions. **C:** Serial dilution growth assays comparing growth of the *tcb1/2/3*Δ query strain to single *nvj1*Δ and quadruple *nvj1*Δ *tcb1/2/3*Δ deletions, without and with copper-titratable *prCUP1-Tcb3-*Δ*SMP-GFP* rescue plasmid on substrates with varying $CuSO_2$ concentrations. **D:** Live FM of cells expressing Tcb3-GFP from the endogenous locus and of *nvj1*Δ *tcb1/2/3*Δ cells with *prCUP1-Tcb3-*Δ*SMP-GFP* after 2 days of growth on plates with varying $CuSO_2$ concentrations, thus comparable to the serial dilution growth assays. Fluorescent images in B and D are adjusted to the same contrast and brightness levels. Scale bars: 2 *µ*m in B and D.

Supplemental Figure S3. Related to Figure 5.

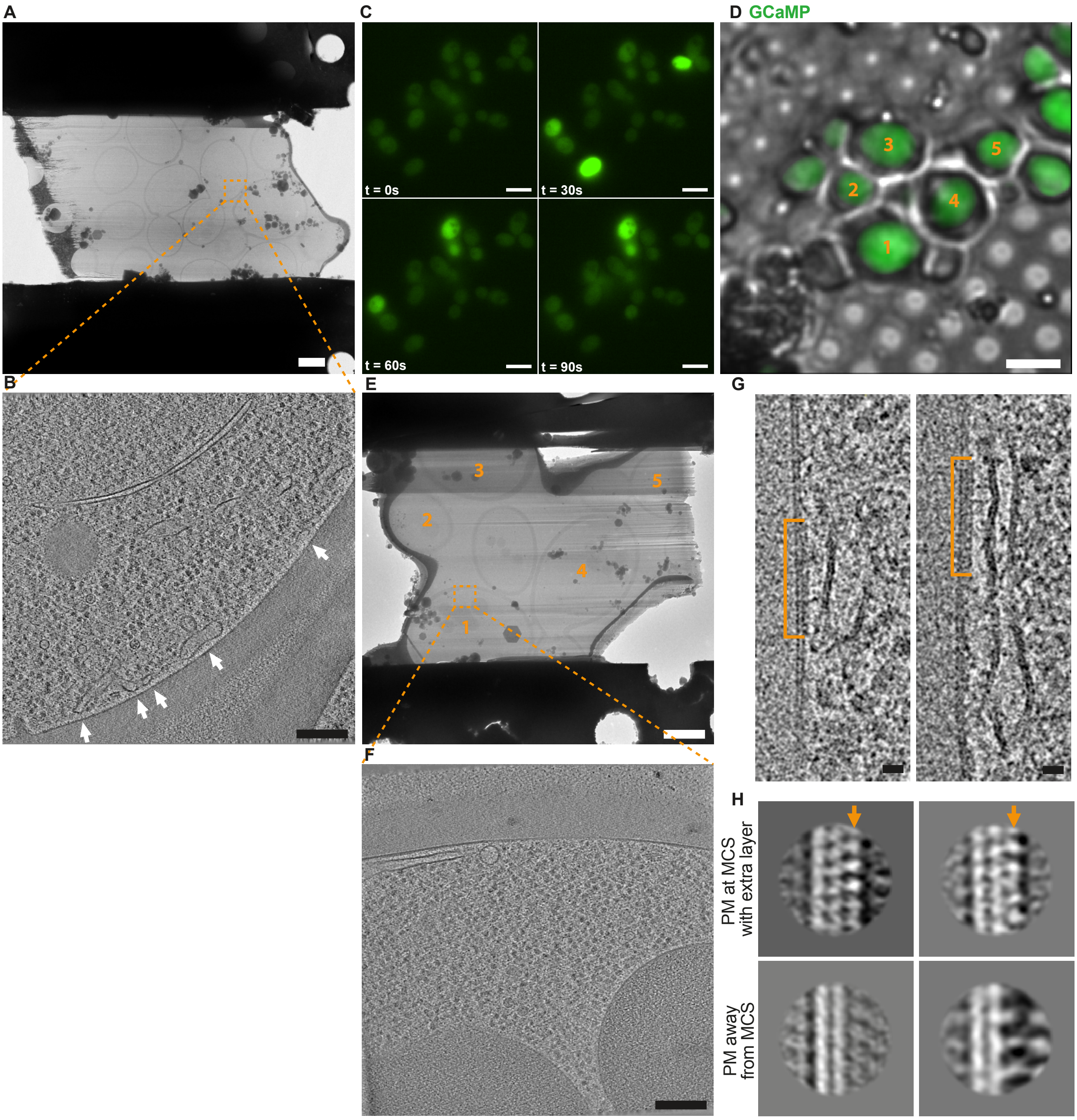

Supplemental Figure S3. Related to Figure 5. Cryo-CLEM for targeting cells with high cytosolic Ca^{2+} by cryo-FIB milling and cryo-ET

A: Overview cryo-EM image of a lamella prepared from untreated *scs2/22*Δ *ist2*Δ cells. Orange dashed square indicates the field of view of the electron cryo-tomogram shown in B. **B:** Virtual slice through electron cryo-tomogram acquired at the lamella position indicated in A. Left image in Figure 5A is from the same electron cryo-tomogram. White arrows indicate tubular cER cisternae. **C:** Live FM of a group of *scs2/22*Δ *ist2*Δ cells expressing GCaMP. GCaMP signal (green) imaged at different time points (0s, 30s, 60s and 90s) after exposure to 200 mM $CaCl_2$. **D:** Cryo-FM image of *scs2/22*Δ *ist2*Δ cells expressing GCaMP; overlay of green and bright field signals. Cells that display strong GCaMP signals, and that are visible in the cryo-FIB milled lamella (E) prepared from this group of cells, are labeled by matching numbers 1 – 5 in both D and E. **E:** Cryo-EM overview of FIB-milled lamella generated from the group of cells shown in D; labeled by matching numbers. Orange dashed square indicates the field of view of the electron cryo-tomogram shown in F. Note that this image corresponds to the image in Figure 5B, but is shown here without overlaid GCaMP signal. **F:** Virtual slice through electron cryo-tomogram acquired at the lamella position indicated above in cell number 1. Magnified views of virtual slices from this tomogram are shown in Figure 5C left and middle. **G:** Virtual slices of electron cryo-tomograms of cells displaying strong GCaMP signals, examples showing an extra density layer (orange brackets) in addition to those shown in Figure 5C. Left panel is a different slice from the same tomogram as shown in middle panel of Supplemental Figure S4A. **H:** 2D class averages from subtomogram averaging of PM within ER-PM contact with extra layer (top images), and PM outside of ER-PM contact (bottom). Class averages to the left and right correspond to tomogram shown in left and right image of G, respectively. In class average images, extracellular PM leaflets are facing left, cytosolic leaflets are facing right. Scale bars: 2 μm in A and E, 200 nm in B and F, 5 μm in C and D, and 20 nm in G.

Supplemental Figure S4. Related to Figures 5 and 6.

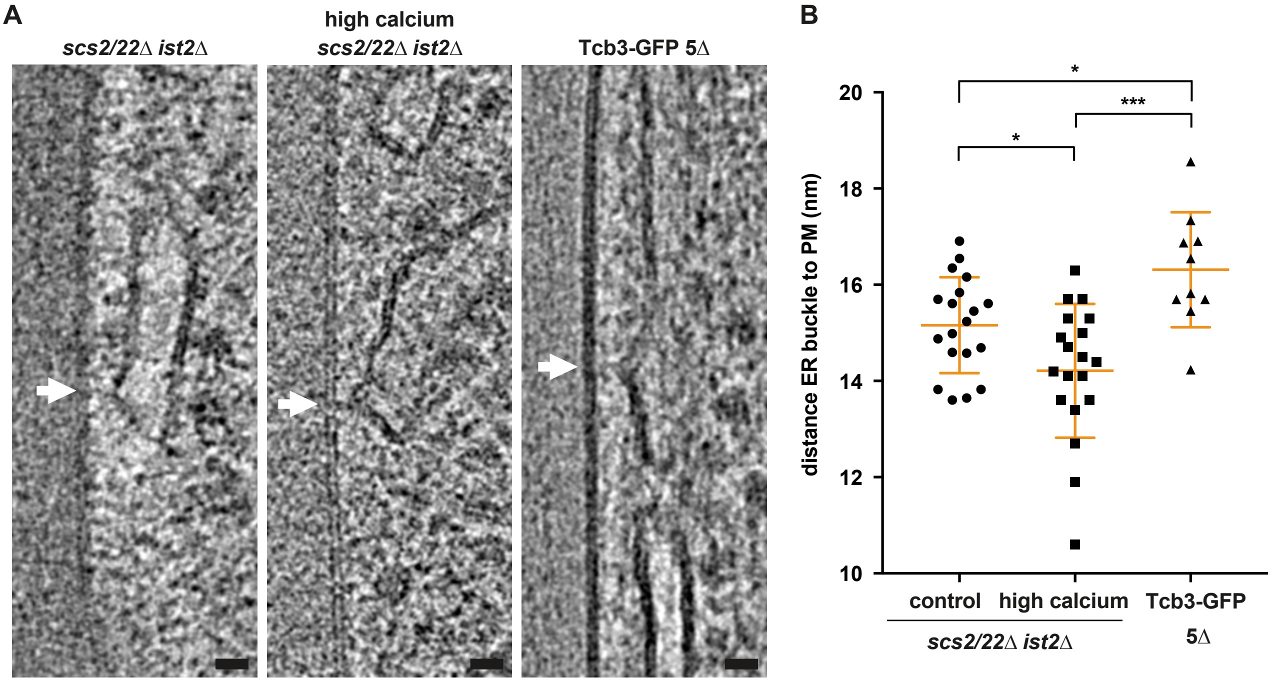

Supplemental Figure S4. Related to Figures 5 and 6. The cER locally buckles towards the plasma membrane

A: Virtual slices through electron cryo-tomograms of three different conditions; untreated *scs2/22*Δ *ist2*Δ cells, Ca^{2+}-treated *scs2/22*Δ *ist2*Δ cells displaying strong GCaMP signal, and 5Δ cells expressing galactose-induced Tcb3-GFP. Arrows indicate local buckles of the cER. Middle panel is a different slice through the same tomogram as shown in left panel of Supplemental Figure S3G. Scale bars: 20 nm. **B:** Distances between the buckling cER and the plasma membrane, measured locally at positions of buckling. Orange lines represent mean and SD (untreated vs. treated: P=0.0213, untreated vs. 5Δ cells: P=0.0192, treated vs. 5Δ cells: P=0.0004).

Supplemental Figure S5. Related to Figure 6.

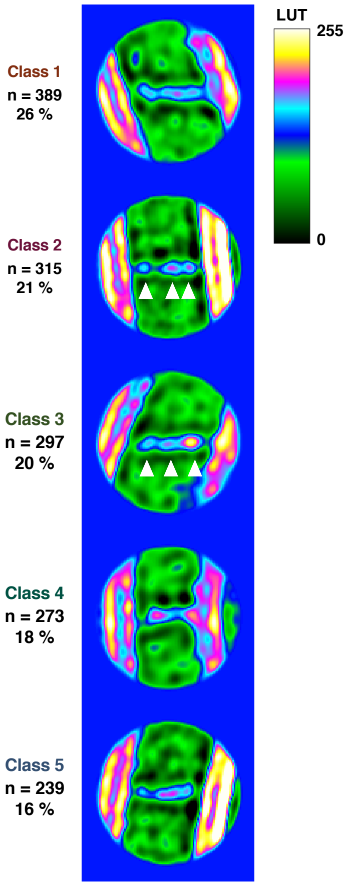

Supplemental Figure S5. Related to Figure 6. 2D class averages reveal linear density organization of rod-like particles

2D class averages of subvolumes containing bridging particles as shown in Figure 6F, but the grey scale was converted to heat map for better visibility of density variations, indicated by arrowheads in classes 2 and 3. LUT indicates color corresponding to gray scale values. Number of particles per class (n), as well as percentage of total particle set, are indicated as in Figure 6F.
